# Supplementary material for: A systematic review and meta-analysis of physical exercise non-adherence and its determinants among type 2 diabetic patients in Ethiopia
Source: PLoS One. 2024 Dec 4;19(12):e0314389. doi: 10.1371/journal.pone.0314389 (PMC11616846; doi:10.1371/journal.pone.0314389)
Supplement: S1 File — (DOCX) [file pone.0314389.s001.docx]

| **Section and Topic** | **Item #** | **Checklist item** | **Location where item is reported** |
| --- | --- | --- | --- |
| **TITLE** | | |  |
| Title | 1 | A systematic review and meta-analysis of physical exercise non-adherence and its determinants among type 2 diabetic patients in Ethiopia | 1 |
| **ABSTRACT** | | |  |
| Abstract | 2 | **Abstract**  **Introduction**: physical exercise non-adherence can lead to overweight and obesity which can be one of the strongest risk factors for Type 2 diabetes mellitus. Therefore, the current study was conducted to determine the pulled prevalence and its determinants of non-adherence to physical activity recommendations among type 2 diabetes adult patients in Ethiopia.  **Methods**: Studies were searched systematically using International databases from PubMed, Google Scholar, Cochrane Library, Embase, and CINAHL. The quality of searched articles assessed using the New Castle Ottawa scale for a cross-sectional study design. Statistical analysis was performed using STATA version 14 and systemic review carried out using a random effect model method. The Preferred Reporting Item for Systematic Review and meta-analyses (PRISMA) guideline was followed for reporting results.  **Result**: From the total 1711 records screened, 7 studies with 3437 participants who fulfilled the inclusion criteria were included in this systematic review. The estimated pooled prevalence of job satisfaction of health care professionals in Ethiopia was 50.59%. Being female (OR=1.27,95% CI (1.82, 1.97)), primary level education (OR=1.19, 95% CI (1.01, 1.39)) and rural residency (OR=4.87, 95% CI (2.80, 8.48)) were significantly associated with exercise non-adherence  **Conclusion**: More than half diabetes patients had physical exercise non-adherence. Strategies such as emotional support, health education, and emphasis for rural diabetic patients can improve the problem of non-adherence.  **Keywords**: physical exercise non-adherence, type 2 diabetes patients, determinant factors, Ethiopia | 2 |
| **INTRODUCTION** | | |  |
| Rationale | 3 | Sixty percent of patients with type 2 diabetes are not increasing their level of exercise following their diagnosis [9]. Despite this fact the rates of non-adherence to exercise recommendations are still as high as 70%. Ethiopia takes the highest percentage 64.3% of diabetic patients had poor adherence to physical activity .  Most of the predictors of non-adherence to exercise were lack of information, the perception that exercise exacerbated their illness, lack of an exercise partner, Lower income and increasing age, being busy, laziness and coexisting diseases were most of the predictors of non-adherence of exercise recommendation.  In the past years solutions have been tried to correct non-adherence problems among patients with type-2 DM. Health professionals’ have been struggling to encourage patients by educating the importance of lifestyle modification on regular exercise; but still we do not achieve the desired outcome.  The finding of these different studies reports that there was high variability in the prevalence of non-adherence to physical activity recommendations among type 2 diabetes adult patients in Ethiopia  Up to the knowledge of the researcher, there was no systematic review done in Ethiopia related to this area of study. The finding of this study will be used as an input to policymakers in non-adherence to physical activity recommendations among type 2 diabetes adult patients in Ethiopia. Therefore, the current study was conducted to determine the pulled prevalence and its determinants of non-adherence to physical activity recommendations among type 2 diabetes adult patients in Ethiopia. | 3-4 |
| Objectives | 4 | prevalence of physical exercise non-adherence and its determinants among type 2 diabetic patients in Ethiopia | 4 |
| **METHODS** | | |  |
| Eligibility criteria | 5 | Quantitative studies that were reported the prevalence of overall physical exercise non-adherence of type 2 diabetic patients, master's thesis, and dissertations where include in the study whereas Qualitative study design, single case study research reports, not fully accessed articles, and poor methodological quality excluded from the analysis.. | 7 |
| Information sources | 6 | We searched on PubMed, Google (for grey literature), Google Scholar, Web of Science, and Cochrane Library databases for studies reporting physical exercise non-adherence | 6 |
| Search strategy | 7 | The known and international databases (PubMed, Scopus, Web of Science, and Cochran library) and searching engines (goggle and Google scholar) were used to locate research articles on the prevalence of physical exercise non-adherence type two diabetic patients in Ethiopia . The string for searching was developed using “AND” and “OR” Boolean operators with the keywords extracted from the Medical Subject Headings (MeSH) database. The search strategy based the research question of this review and utilized the CoCoPop (Co=Condition, Co=Context, Pop=Population) for prevalence and PEO, (Pop=Population, E= exposure, O=outcome interest) for determinant factors.  The article locating strategy was through “physical exercise non-adherence” OR "physical inactivity" OR "exercise none-adherence" OR "prevalence physical exercise non-adherence” AND "type two diabetes" OR “diabetes " OR "insulin resistance diabetes" AND "Ethiopia ". This searching strategy primarily aimed to trace all reviewed (published) and unpublished primary studies. The list of all retrieved primary articles and systematic review and meta-analyses reference were also screened or cross referenced to get extra studies. The sources of information range from electronic data bases to direct contact with principal investigator if mandatory. The first search through Pub Med, Cochran library, Scopus, Web of Science, Google, and Google scholar was done in April, 2023. The final search for updating was conducted from June5/ 2023 to June 29/ 6/2023.Publication date was used as filter mechanism in which articles published from January 2013 to June 29/2023 included to the current systematic Review and Meta-analysis study to generate the most recent evidence to the scientific community. | 6 |
| Selection process | 8 | Eligible research articles were screened by their title (Ti), Abstract (Ab), and full-text. Two reviewers independently reviewed the included articles. | 6 |
| Data collection process | 9 | The data were extracted by data abstraction format using the Microsoft excel spreadsheet. The format was developed by two reviewers and piloted for its clarity, aim, consistency, and depth of the contents. Simple and consistent codes of response were used. Then the reviewers independently reviewed and extract data from each eligible study. The information such as authors, publication year, region of the study, design, methodological quality, population, study setting, sample size, method of data collection, and statistical analysis were extracted from the studies. | 8 |
| Data items | 10a | Articles that have clearly defined outcome variables were included | 8 |
|  | 10b | The variables that have direct effect for the occurrence of physical exercise non-adherence were identified | 8 |
| Study risk of bias assessment | 11 | Articles were assessed for quality score using the New Castle Ottawa Scale adapted from cross-sectional, a score of ≥ 7 out of 10 was considered a high-quality score. Two authors assessed the quality of each paper. The reviewers compared the quality of the appraisal scores and resolved inconsistencies prior to calculating the final appraisal score. | 7-8 |
| Effect measures | 12 | The odds ratio (or), logor, and standard error or (SeOr) were used presentation of results. | 9 |
| Synthesis methods | 13a | Quantitative analysis were employed. | 6 |
|  | 13b | The crud odds ratio(COR) or adjusted odds ratio(AOR) were used for data presentation or evidence synthesis. | 9 |
|  | 13c | PRISMA flow chart, forest plot, and funnel plot were used to present visually displayed data | 9 |
|  | 13d | The Cochrane Q statistics and (I2) were used to assess the heterogeneity status of the included studies. | 9 |
|  | 13e | random-effects model was employed to estimate the analysis | 9 |
|  | 13f | The quantitative synthesis was employeed |  |
| Reporting bias assessment | 14 | Begg’s and/or Egger’s test was computed to detect publication bias. | 9 |
| Certainty assessment | 15 | The pooled summary effect size of the study was estimated. | 9 |
| **RESULTS** | | |  |
| Study selection | 16a | In this systematic review and meta-analysis study a total of 1711 articles related to the prevalence of exercise non-adherence in Ethiopia were identified using electronic databases and searching engine websites. Among overall articles found 1201 were removed for being irrelevance and duplicated and the other 231 and 113 were removed for not being ineligible (study design and title difference) by automation tools and other reasons respectively. The remaining 74 articles were eligible for screening. Of these screened 51 papers were excluded due to region of study or not conducted in Ethiopia and target population difference (those articles conducted among typ1 diabetes). With further screening 22 articles were sought for retrieval and 8 were not retrieved by one and the other reason. Moreover, 14 research articles were assessed for eligibility to be included for the review process, but with the outcome of interest and measurement tool ambiguity a total of 7 articles were excluded. Finally, 7 original research articles were incorporated with the systematic review and meta-analysis (Figure 1). | 10 |
|  | 16b | Studies were excluded because of outcome interest and not full text articles. | 10 |
| Study characteristics | 17 | All research articles included in this systemic review were done by cross-sectional study design and published from January 2013 to June 29/2023. Seven published studies with 3437 participants were included to determine the pooled prevalence of physical exercise non-adherence among type 2 diabetes patients. All articles were conducted with a cross-sectional study design with the smallest prevalence was from Oromia (11.9%) [31] and the largest prevalence was from Amhara 73.6% [33] regional state of Ethiopia. On the contrary, the largest sample size was from Oromia region (1191) [30] whereas the smallest sample size was from Amhara (302) [34]regional state of Ethiopia. This review includes three studies from the Amhara regional [32-34] and four studies from Oromia [13, 29-31] regional state of Ethiopia (Table 1). |  |
| Risk of bias in studies | 18 | Intermes of qualtity all included studies are good quality | 10 |
| Results of individual studies | 19 | Table 1: Summary of the prevalence of physical exercise non-adherence among seven studies of included in the systematic review and meta-analysis   \| Author/year of publication \|  \| Region \| Sample method \| Sample size \| Outcome \| Prevalence \| Response rate (%) \| Quality \| \| --- \| --- \| --- \| --- \| --- \| --- \| --- \| --- \| --- \| \| Debalke et al.[29] \| 2022 \| Oromia \| systematic random sampling \| 392 \| 243 \| 38 \| 92.9 \| 9 \| \| Zenu el. al.[30] \| 2023 \| Oromia \| mult-stage sampling \| 1191 \| 729 \| 61.2 \| 93.3 \| 9 \| \| Abate et al. [33] \| 2020 \| Amhara \| systematic random sampling \| 576 \| 450 \| 73.6 \| 99.3 \| 8 \| \| Enyew et. Al.[34] \| 2023 \| Amhara \| systematic random sampling \| 302 \| 93 \| 72 \| 98 \| 7 \| \| Edmealem. Et.al.[32] \| 2020 \| Amhara \| systematic random sampling \| 332 \| 110 \| 33.1 \| 91.2 \| 8 \| \| Negra et.al [13] \| 2020 \| oromia \| simple random sampling \| 322 \| 206 \| 64.3 \| 100 \| 6 \| \| Tamirat et.al [31] \| 2014 \| oromia \| systematic random sampling \| 322 \| 41 \| 11.9 \| 99.1 \| 8 \| | 10 11 |
| Results of syntheses | 20a | The risk of bias was assed using Begg’s and/or Egger’s test. | 9 |
|  | 20b | **Pooled prevalence of physical exercise non-adherence among type two diabetes in Ethiopia**  The prevalence of physical exercise non-adherence range from 11.7% from Oromia [31] region to 73.6% among type 2 diabetes patients in the Amhara [33] regional states of Ethiopia. The pooled prevalence of physical exercise non-adherence among type 2 diabetes patients was 50.59% with 95% CI (24.59-66.58) based on the DerSimonian-Laird random effect model analysis (figure 2).  **Assessment of heterogeneity**  In this systematic review, subgroup analysis was done to assess the heterogeneity, so that the p- value and I^2^ statistics where used to assess the heterogeneity between studies. The analysis result showed that the source of heterogeneity is not due to region (p=0.0001 I^2 =^100). The lowest pooled prevalence of physical exercise non-adherence of type 2 diabetes patients was found in Oromia region at 43.85% (95% CI 18.66 - 69.04) and the highest pooled prevalence in Amhara regional state of Ethiopia 59.57 % (95% CI (35.03 – 84.10) (Figure 3).  The source of heterogeneity further assessed using study year, sampling technique, or study setting to identify the reason for variation among studies but none of them are the source of heterogeneity (Table 2).  **Meta-regression**  Furthermore, of subgroup analysis, univariate meta-regression is carried out with sample size, and publication year for possible heterogeneity. The result of the analysis indicates that none of them significantly affected heterogeneity between studies.  **Publication bias**  The funnel plot was done to show to check the publication bias. This study has no publication bias since it is symmetrically distributed. Furthermore, statistically, Begg's test and Egger test were done with p-value =0.42 which showed that there no publication bias (Fig 3).  **Sensitivity analysis**  A sensitivity analysis was performed after observing lower and higher value on the review to show the effect of one study on the overall pulled summary effect. However, the analysis result of the sensitivity test using the random-effects model indicated that no single affected the overall estimate (Figure 5).    **The pooled analysis of determinant factors**  **The effect of sex of participants**  Being female sex was a contributing factors for physical exercise non-adherence with 1.27 times increase the overall pooled prevalence of physical exercise non-adherence among type 2 diabetes patients (OR=1.27,95% CI (1.82, 1.97)) (Figure 6).  **The effect of being primary education**  Being primary education was a contributing factors for physical exercise non-adherence with 1.19 times increase the overall pooled prevalence of physical exercise non-adherence among type 2 diabetes patients (OR=1.19, 95% CI (1.01, 1.39)) (Figure 8).  **The effect of rural residency**  Being rural residency was a contributing factors for physical exercise non-adherence with 4.87 times increase the overall pooled prevalence of physical exercise non-adherence among type 2 diabetes patients (OR=4.87, 95% CI (2.80, 8.48)) (Figure 8). | 11-13 |
|  | 20c | **Heterogeneity and Publication bias of the included studies for being female sex**  As stated figure 6 showed that the overall heterogeneity test (I^2^) on the effect of being female sex was 0.0% with the p-value 0.977, using random effect model to adjust the observed variability. This heterogeneity test indicates there is observed variability across the included studies.  **Heterogeneity and Publication bias of the included studies for primary education**  As stated above figure 8, the overall heterogeneity test (I^2^) on the effect of being primary education was 0.0% with the p-value 0.548, using random effect model to adjust the observed variability. This heterogeneity test indicates there is observed variability across the included studies.  **Heterogeneity and Publication bias of the included studies for rural recidency**  As stated above figure 10, the overall heterogeneity test (I^2^) on the effect of being rural recidency was 20.2% with the p-value 0.057, using random effect model to adjust the observed variability. This heterogeneity test indicates there is observed variability across the included studies. | 11-13 |
|  | 20d | Regarding the publication of being female sex bias, the funnel plot showed that symmetrical graphic presentation which indicates no publication bias (Figure 7). Furthermore, statistically, Egger test was done with p = 0.31 which showed that there no publication bias. Regarding the publication bias of primary education, the funnel plot showed that symmetrical graphic presentation which indicates no publication bias (Figure 9). Furthermore, statistically, Egger test was done with p = 0.31 which showed that there no publication bias. Regarding the publication bias, of rural recidency the funnel plot showed that symmetrical graphic presentation which indicates no publication bias (Figure 11). Furthermore, statistically, Egger test was done with p = 0.134 which showed that there no publication bias. |  |
| Reporting biases | 21 | Publication bias  The funnel plot was done to show to check the publication bias. This study has no publication bias since it is symmetrically distributed. Furthermore, statistically, Begg's test and Egger test were done with p-value =0.42 which showed that there no publication bias (Fig 3). | 12 |
| Certainty of evidence | 22 | In this systematic and meta-analysis study we had estimated the pooled prevalence of exercise non-adherence of type two patient factors were associated such as , being female sex, primary education , and rural residency | 12-13 |
| **DISCUSSION** | | |  |
| Discussion | 23a | In this systematic review and meta-analysis, the pooled prevalence of physical exerciser non-adherence among diabetes patients was 50.59% with 95% CI (24.59-66.58). This finding was in line with study conducted in Botswana (52%) [37]. This figure showed that more than half of the type 2 diabetes participant was non-adherence to physical exercise. The main possible justification might be due to lack of education/ information about the importance physical exercise adherence. Evidence also support that exercise non-adherence mattered by due to wrong perception that exercise can aggravate the prognosis’s diabetic [38]. This finding is lower than the study conducted in West Africa 13% [39]. The possible justification might be due to the study conducted in West Africa include many countries compared to the current study which might lead to decrease the pooled prevalence of physical inactivity. The finding also supported by the study conducted USA [40, 41], Australia [42, 43].  In this meta-analysis the pooled of determinant such as being female sex, primary education and being rural residency were significantly associated physical exercise non-adherence among type 2 diabetes patients. Therefore, being female was 1.27 times increase the overall pooled prevalence of physical exercise non-adherence among type 2 diabetes patients compared to male participants. Even though there was no similar study, the finding was supported by the study conducted in Botswana [37]. This might be due to lack of lack of exercise partner, lack of motivation, and lack of community support for females [38].  Being primary education was a contributing factor for physical exercise non-adherence with 1.19 times increase the overall pooled prevalence of physical exercise non-adherence among type 2 diabetes patients. This finding was supported by the study conducted Switzerland [44]. This because education can increase individual person information about the importance life style modification including physical exercise in the management of chronic disease [45].  Being rural residency was a contributing factors for physical exercise non-adherence with 4.87 times increase the overall pooled prevalence of physical exercise non-adherence among type 2 diabetes patients compared to urban residency. This finding was supported by the study conducted in Pakistan [46]. Although rural diabetes are physically more active than participants urban areas they might have no regular schedule to physical exercise due to many reasons such as health access to health information about physical exercise non-adherence [47]. |  |
|  | 23b | The studies included in this review were cross-sectional as a result; the outcome variable might be affected by other confounding variables. Moreover, some regions were not incorporated because of lack of research may lead to an underestimate of this review.. | 15 |
|  | 23c | The study has an implication for policymakers and clinicians to plan and implement possible interventions to prevent the occurrence and severe outcome exercise non-adherence . | 15 |
|  | 23d | Improving strategies such as empowering women, preparing leaflets about diabetic information and organizing health education about the scientific merit of exercise for diabetic patients is among the best modalities to improve the problem exercise non-adherence. In addition, special attention should be given for rural diabetic patients since they had poor exercise adherence compared to the urban diabetic patients | 15 |
| **OTHER INFORMATION** | | |  |
| Registration and protocol | 24a | The review protocol has been registered in the international prospective register of systematic reviews (PROSPERO) with registration number PROSPERO: CRD42023416724.. | 6 |
|  | 24b | The review protocol can be accessed via online databases.. |  |
|  | 24c | Further amendments may /not needed. |  |
| Support | 25 | The Authors did not receive any fund for this particular study. |  |
| Competing interests | 26 | There is no competing of interest. |  |
| Availability of data, code and other materials | 27 | The data extracted were analysed and included in the result. |  |

*From:*  Page MJ, McKenzie JE, Bossuyt PM, Boutron I, Hoffmann TC, Mulrow CD, et al. The PRISMA 2020 statement: an updated guideline for reporting systematic reviews. BMJ 2021;372:n71. doi: 10.1136/bmj.n71

For more information, visit: <http://www.prisma-statement.org/>
