# Supplementary material for: A systematic review and meta-analysis of physical exercise non-adherence and its determinants among type 2 diabetic patients in Ethiopia
Source: PLoS One. 2024 Dec 4;19(12):e0314389. doi: 10.1371/journal.pone.0314389 (PMC11616846; doi:10.1371/journal.pone.0314389)
Supplement: S2 File — (DOCX) [file pone.0314389.s002.docx]

S2 File : Supplementary file for searching strategies

| Bock 1 | | |  | |
| --- | --- | --- | --- | --- |
| “physical exercise non-adherence” [Mesh]  “physical inactivity” [Mesh]  “exercise none-adherence” [Mesh]  “exercise adherence” [Mesh]  “prevalence physical exercise non-adherence” [Mesh] | | | **Free text search in the title and abstract**  Physical inactivity*  Exercise non-adherence*  Physical exercise non-adherence* | |
| Block 2 | | |  | |
| “Diabetes”[ Mesh]  “Type two diabetes “[Mesh]  “insulin resistance diabetes” [Mesh] | | | Diabetes*  Type two diabetes *  insulin resistance diabetes* | |
| Data base |  | Searching strings | | Searching result |
|  | PubMed/Medline | The string for searching was developed using “AND” and “OR” Boolean operators with the keywords extracted from the Medical Subject Headings (MeSH) database. The search strategy was based on the research question of this review and utilized CoCoPop (Co=Condition, Co=Context, Pop=Population) for prevalence and PEO (Pop=Population, E= exposure, O=outcome interest) for determinant factors.  For PubMed/Medline we had used the following searching Boolean operators.  ((((((((physical exercise non-adherence) OR (physical inactivity)) OR (exercise none-adherence)) OR (exercise adherence)) OR (prevalence physical exercise non-adherence)) AND (type two diabetes)) OR (diabetes)) OR (insulin resistance diabetes)) AND (Ethiopia) | | 1703 |
|  | Embase | Based on modeled search strategy design for Embase using Ttile(Ti) and Abstract (Ab) | | 22 |
|  | Cochran library | Based on modeled search strategy design for Embase | | 77 |
|  | Scopus | Based on modeled search strategy design for Cochran using Ttile(Ti) and Abstract (Ab) | | 8 |
| Searching engines | Google advance | Using title | | 33 |
|  | Google Scholar | Using title | | 84 |
|  | OpenGrey, | Using title | | 72 |
|  | ProQuest | Using title | | 697 |

| For all above searched articles  Total articles searched (3500) |
| --- |

| Articles removed before screening (due to duplication, ineligible by auto machine tool and for other reason ) | No of article |
| --- | --- |
|  | 2608 |
| Screened record | 88 |
| Reports sought for retrieval | 22 |
| Reports assessed for eligibility | 8 |
| Studies included and reported in the final review | 7 |
