# Supplementary material for: A systematic review and meta-analysis of physical exercise non-adherence and its determinants among type 2 diabetic patients in Ethiopia
Source: PLoS One. 2024 Dec 4;19(12):e0314389. doi: 10.1371/journal.pone.0314389 (PMC11616846; doi:10.1371/journal.pone.0314389)
Supplement: S3 File — (DOCX) [file pone.0314389.s003.docx]

**S4 File: An explanation of how missing data were handled.**

As it is known missing data results in decreased precision and possibly biased effect estimates of single studies. However, there was no missing among the included primary articles in our systematic review and meta-analysis study because we included them all and a pooled estimate was generated. This is because we have an extensive search of articles in order not to miss data by making our searching and pre-define criteria for including and excluding the primary articles clear and precise. We have used a standardized data extraction form for each single study included and also conducted a robust inspection of our data before formal analysis took over. In addition, we have conducted sensitivity and trim and fill analysis to detect and adjust potential publication bias.
