# Supplementary material for: A systematic review and meta-analysis of physical exercise non-adherence and its determinants among type 2 diabetic patients in Ethiopia
Source: PLoS One. 2024 Dec 4;19(12):e0314389. doi: 10.1371/journal.pone.0314389 (PMC11616846; doi:10.1371/journal.pone.0314389)
Supplement: S1 Table — (DOCX) [file pone.0314389.s004.docx]

S1 Table : The modified version of Newcastle-Ottawa Scale for cross-sectional studies

| Included studies | Selection criteria point | | | | Comparability criteria point | Outcome criteria point | | Total /9 |
| --- | --- | --- | --- | --- | --- | --- | --- | --- |
|  | Q1s | Q2s | Q3s | Q4s | Q1c | Q1O | Q2O |  |
| Debalke et al. | 1 | 1 | 1 | 2 | 2 | 1 | 1 | 9 |
| Zenu el. al. | 1 | 1 | 1 | 2 | 2 | 1 | 1 | 9 |
| Abate et al. | 1 | 1 | 1 | 0 | 2 | 0 | 1 | 8 |
| Enyew et. al. | 1 | 0 | 0 | 2 | 2 | 1 | 1 | 7 |
| Edmealem. et.al. | 1 | 1 | 1 | 0 | 2 | 1 | 1 | 8 |
| Negra et.al. | 1 | 0 | 1 | 0 | 2 | 0 | 1 | 7 |
| Tamirat et al | 1 | 1 | 0 | 2 | 2 | 1 | 1 | 8 |
| Eshet et al. | 1 | 1 | 1 | 0 | 0 | 0 | 0 | 3 |
| Korya et al | 1 | 1 | 1 | 0 | 0 | 0 | 0 | 3 |

Q1s: Representativeness of the cases, Q2s: Sample size: Q3s: Non-Response rate Q4s: Ascertainment of the screening/surveillance tool, Q1c: potential confounders were investigated, Q1O: Assessment of the outcome, Q2O: is statistical test used to analyze the data
