# Supplementary material for: A systematic review and meta-analysis of physical exercise non-adherence and its determinants among type 2 diabetic patients in Ethiopia
Source: PLoS One. 2024 Dec 4;19(12):e0314389. doi: 10.1371/journal.pone.0314389 (PMC11616846; doi:10.1371/journal.pone.0314389)
Supplement: S2 Table — (DOCX) [file pone.0314389.s005.docx]

| author | Publication  year | region | study  design | study setting | sampling method | sample  size | prevalence | Date of data extraction | Name data extractor | Eligibility |
| --- | --- | --- | --- | --- | --- | --- | --- | --- | --- | --- |
| Debalke et al. | 2022 | Oromia | cross-sectional | hospital | systematic random sampling | 392 | 38 | 5/ 2023 to June 29/ 6/2023 | HKA, SST | Eligibility |
| Zenu el. al. | 2023 | Oromia | cross-sectional | community | mult-stage sampling | 1191 | 61.2 | 5/ 2023 to June 29/ 6/2023 | HKA, SST | Eligibility |
| Abate et al. | 2020 | Amhara | cross-sectional | community | mult-stage sampling | 576 | 73.6 | 5/ 2023 to June 29/ 6/2023 | HKA, SST | Eligibility |
| Enyew et. Al. | 2023 | Amhara | cross-sectional | hospital | systematic random sampling | 302 | 72 | 5/ 2023 to June 29/ 6/2023 | HKA, SST | Eligibility |
| Edmealem. Et.al. | 2020 | Amhara | cross-sectional | hospital | systematic random sampling | 332 | 33.1 | 5/ 2023 to June 29/ 6/2023 | HKA, SST | Eligibility |
| Negra et.al | 2020 | Oromia | cross-sectional | hospital | systematic random sampling | 322 | 64.3 | 5/ 2023 to June 29/ 6/2023 | HKA, SST | Eligibility |
| Tamirat et.al | 2014 | Oromia | cross-sectional | hospital | systematic random sampling | 322 | 11.9 | 5/ 2023 to June 29/ 6/2023 | HKA, SST | Eligibility |

S2 Table: A table of all data extracted from the primary research sources for the systematic review and/or meta-analysis

**Significant factors of associated variables for exercise nonadherence**

**Being Female diabetes**

| S/N | publication year | study design | sample size | AOR | UBCI | LBCI | logor | selogor | Name of Data extractor | Date of data extraction | Eligibility confirmation |
| --- | --- | --- | --- | --- | --- | --- | --- | --- | --- | --- | --- |
| Debalke et al. | 2022 | cross-sectional | 392 | 0.612903226 | 1.63 | 3.8 | 0.396722279 | 0.553571429 | HKA,SST | 5/ 2023 to June 29/ 6/2023 | Eligible |
| Zenu el. al. | 2023 | cross-sectional | 1191 | 1.577319588 | 2.4 | 5.6 | 0.556302501 | 0.816326531 | HKA,SST | 5/ 2023 to June 29/ 6/2023 | Eligible |
| Abate et al. | 2020 | cross-sectional | 576 | 2.787878788 |  |  |  |  | HKA,SST | 5/ 2023 to June 29/ 6/2023 | Eligible |
| Enyew et. Al. | 2023 | cross-sectional | 302 | 2.571428571 | 1.15 | 3.10 | 0.11058971 | 0.49744898 | HKA,SST | 5/ 2023 to June 29/ 6/2023 | Eligible |
| Edmealem. Et.al. | 2020 | cross-sectional | 332 | 0.494768311 | 0.87 | 2.19 | 0.139879086 | 0.336734694 | HKA,SST | 5/ 2023 to June 29/ 6/2023 | Eligible |
| Negra et.al | 2020 | cross-sectional | 322 | 1.801120448 | 1.68, | 4.34 | 0.431363764 | 0.678571429 | HKA,SST | 5/ 2023 to June 29/ 6/2023 | Eligible |
| Tamirat et.al | 2014 | cross-sectional | 322 | 0.13507378 | 1.63 | 3.80 | 0.396722279 | 0.553571429 | HKA,SST | 5/ 2023 to June 29/ 6/2023 | Eligible |
| Being primary educational level | | | | | | | | | | | |
| Authors | publication year | study design | sample size | AOR | UBCI | LBCI | logor | selogor | Name of Data extractor | Date of data extraction | Eligibility confirmation |
| Debalke et al. | 2022 | cross-sectional | 392 |  |  |  |  |  | HKA,SST | 5/ 2023 to June 29/ 6/2023 | Eligible |
| Zenu el. al. | 2023 | cross-sectional | 1191 | 0.833 | 0.8 | 1.83 | -0.0793 | 0.262755102 | HKA,SST | 5/ 2023 to June 29/ 6/2023 | Eligible |
| Abate et al. | 2020 | cross-sectional | 576 | 0.97 | 0.42 | 2.26 | -0.0132 | 0.477040816 | HKA,SST | 5/ 2023 to June 29/ 6/2023 | Eligible |
| Enyew et. Al. | 2023 | cross-sectional | 302 |  |  |  |  |  | HKA,SST | 5/ 2023 to June 29/ 6/2023 | Eligible |
| Edmealem. Et.al. | 2020 | cross-sectional | 332 |  |  |  |  |  | HKA,SST | 5/ 2023 to June 29/ 6/2023 | Eligible |
| Negra et.al | 2020 | cross-sectional | 322 |  |  |  |  |  | HKA,SST | 5/ 2023 to June 29/ 6/2023 | Eligible |
| Tamirat et.al | 2014 | cross-sectional | 322 | 1.6 | 0.04 | 0.38 | 0.2041 | 0.086734694 | HKA,SST | 5/ 2023 to June 29/ 6/2023 | Eligible |
| Being rural residency | | | | | | | | | | | |
| Authors | publication year | study design | sample size | AOR | UBCI | LBCI | logor | selogor | Name of Data extractor | Date of data extraction | Eligibility confirmation |
| Debalke et al. | 2022 | cross-sectional | 392 |  |  |  |  |  | HKA,SST | 5/ 2023 to June 29/ 6/2023 | Eligible |
| Zenu el. al. | 2023 | cross-sectional | 1191 |  |  |  |  |  | HKA,SST | 5/ 2023 to June 29/ 6/2023 | Eligible |
| Abate et al. | 2020 | cross-sectional | 576 | 1.95 | 1.16 | 3.27 | 0.290034611 | 0.211734694 | HKA,SST | 5/ 2023 to June 29/ 6/2023 | Eligible |
| Enyew et. Al. | 2023 | cross-sectional | 302 | 3.19 | 1.44 | 7.05 | 0.503790683 | 1.431122449 | HKA,SST | 5/ 2023 to June 29/ 6/2023 | Eligible |
| Edmealem. Et.al. | 2020 | cross-sectional | 332 | 1.45 | 0.90 | 2.35 | 0.161368002 | 0.369897959 | HKA,SST | 5/ 2023 to June 29/ 6/2023 | Eligible |
| Negra et.al | 2020 | cross-sectional | 322 | 1.1 | 0.71 | 1.77 | 0.041392685 | 0.270408163 | HKA,SST | 5/ 2023 to June 29/ 6/2023 | Eligible |
| Tamirat et.al | 2014 | cross-sectional | 322 |  |  |  |  |  | HKA,SST | 5/ 2023 to June 29/ 6/2023 | Eligible |
