# Supplementary material for: A systematic review and meta-analysis of physical exercise non-adherence and its determinants among type 2 diabetic patients in Ethiopia
Source: PLoS One. 2024 Dec 4;19(12):e0314389. doi: 10.1371/journal.pone.0314389 (PMC11616846; doi:10.1371/journal.pone.0314389)
Supplement: S3 Table — (DOCX) [file pone.0314389.s006.docx]

|  | **Domain of certainty of included articles** | | | | | | **Factor that increase certainty included articles** | | | | **Overall quality** |
| --- | --- | --- | --- | --- | --- | --- | --- | --- | --- | --- | --- |
| Number of studies | Study design | Publication bias | Indirectness | Inconsistency /heterogeneity | Imprecision | Risk bias | Magnitude of effect | Dose response gradient | Effect of confounding variables |  | |
| 7 | Observational studies (evidence of certainty assessment started at low because of the design) | Not serious (No evidence of publication bias based on funnel plot and egger’s test) | Not serious (all studies the outcome variable objectively) | Serious (significant heterogeneity detected) | Not serious (all included studies have good sample size, narrow confidence interval of the estimate) | Serious (few number of studies included, the method employed in the study is cross-sectional, prone to bias) | Large magnitude of effect is observed in this meta-analysis | Not applicable for observational studies | All included studies have  controlled the effect of confounding | Low | |

**Supplementary file Table S4: GRADE score of the final included studies**

Table for description of Categories of quality of evidence based on GRADE

| Rank | Description of the rank |
| --- | --- |
| High ++++ | Further research is very unlikely to change our confidence in the estimate effect |
| Moderate +++ | Further research is likely to have an important impact in our confidence in the estimate of effect and change the estimate |
| Low ++ | Further research is very likely to have an important impact in our confidence in the estimate effect is likely to change the estimate |
| Very low + | Any estimate of effect is very uncertain |

| Grade assessment |
| --- |
| **Study limitations**: If most trials at low ROB=no downgrade for limitations of design; If most trials at unclear ROB= downgrade for limitations of design by 1 level; If most trials at high ROB= downgrade for limitations of design by 2 levels. |
| **Inconsistency**: 1 or 2 levels of downgrade depending on clinical & methodological heterogeneity (PICO), statistical heterogeneity, Confidence interval overlap |
| **Indirectness**: 1 or 2 levels of downgrade depending on whether or not head-to-head comparisons were used |
| **Imprecision**: 1 or 2 levels of downgrade depending on statistical heterogeneity, CI overlap and inclusion of benefits and harms in the CI |
| **Publication bias**: suspected or unsuspected based on completeness of search strategy, formal statistical assessment and SR authors’ comments |
| **Outcome Importance**: not important; important but not critical; critical. Refers to selected outcome and based on importance relative to intervention. i.e. implant failure critical but clinical attachment loss may be important but not |
| **Effect size:** if effect is strong applicable for observational studies RR>2 or <0.5; RR>5 or RR<0.2 |
| **GRADE rating in this review:** High, moderate, low, very low |
| **GRADE reported by SR authors (if provided):** High, moderate, low, very low |
| **SR=Systematic review; ROB=Risk of bias; CI=Confidence interval; OR=Odds ratio; Risk ratio** |
