# Supplementary material for: A systematic review and meta-analysis of physical exercise non-adherence and its determinants among type 2 diabetic patients in Ethiopia
Source: PLoS One. 2024 Dec 4;19(12):e0314389. doi: 10.1371/journal.pone.0314389 (PMC11616846; doi:10.1371/journal.pone.0314389)
Supplement: S4 Table — (DOCX) [file pone.0314389.s007.docx]

**S4 Table:** All studies identified in the literature search, including those that were excluded from the analyses.

| S/N | Author/s(reference | Included OR Excluded | Reasons for exclusion | Published or unpublished | URL if unpublished |
| --- | --- | --- | --- | --- | --- |
| 1 | Debalke et al.[1] | Included |  | Published |  |
| 2 | Zenu el. al.[2] | Included |  | Published |  |
| 3 | Abate et al. [3] | Included |  | Published |  |
| 4 | Enyew et. Al.[4] | Included |  | Published |  |
| 5 | Edmealem. Et.al.[5] | Included |  | Published |  |
| 6 | Negra et.al [6] | Included |  | Published |  |
| 7 | Tamirat et.al [7] | Included |  | Published |  |
| 8 | Eshet et.al [8] | Excluded | No clear outcome interest | Published |  |
| 9 | Koyra et.al [9] | Excluded | No clear outcome interest | Published |  |
| 10 | Akumiah et al [10] | Excluded | Not Ethiopian study | Published |  |
| 11 | Zhu et.al[11] | Excluded | Not Ethiopian study | Published |  |
| 12 | Alhariri et.al[12] | Excluded | Not Ethiopian study | Published |  |
| 13 | Ganiyu et.al [13] | Excluded | Not Ethiopian study | Published |  |
| 14 | Höchsmann et.al [14] | Excluded | Not Ethiopian study | Published |  |
| 15 | Saleh et.al [15] | Excluded | Not Ethiopian study | Published |  |
| 16 | Parajuli et.al [16] | Excluded | Not Ethiopian study | Published |  |
| 17 | Ghimire et. al [17] | Excluded | Not Ethiopian study | Published |  |
| 18 | Woodard et al [18] | Excluded | Not Ethiopian study | Published |  |
| 19 | Broadbent et. al [19] | Excluded | Not Ethiopian study | Published |  |
| 20 | Forhan et.al [20] | Excluded | Not Ethiopian study | Published |  |
| 21 | Mkonka [21] | Excluded | Not Ethiopian study | Published |  |
| 22 | Pandey, A et.al [22] | Excluded | Not Ethiopian study | Published |  |
| 23 | Jadawala et.al [23] | Excluded | Not Ethiopian study | Published |  |
| 24 | Joshi et.al [24] | Excluded | Not Ethiopian study | Published |  |
| 25 | Muhabuura, B et.al [25] | Excluded | Not Ethiopian study | Published |  |
| 26 | Darawad et.al [26] | Excluded | Not Ethiopian study | Published |  |
| 27 | Wabe, Nasir T et.al [27] | Excluded | Not Ethiopian study | Published |  |
| 28 | Abebe et.al [28] | Excluded | Not Ethiopian study | Published |  |
| 29 | Alhaiti, Ali Hassan et. al [29] | Excluded | Not Ethiopian study | Published |  |
| 30 | Afaya et.al [30] | Excluded | Not Ethiopian study | Published |  |
| 31 | Dubasi et.al [31] | Excluded | Not Ethiopian study | Published |  |
| 32 | Rustveld et.al [32] | Excluded | Not Ethiopian study | Published |  |
| 33 | Musenge et al [33] | Excluded | Not Ethiopian study | Published |  |
| 34 | Peng, Xi et al [34] | Excluded | Not Ethiopian study | Published |  |
| 35 | Nam et.al [35] | Excluded | Not Ethiopian study | Published |  |
| 36 | Enikuomehine et.al [36] | Excluded | Not Ethiopian study | Published |  |
| 37 | Muriungi, S.K. and D.M. Ndetei[37] | Excluded | Not Ethiopian study | Published |  |
| 38 | Makhubela, M.et al [38] | Excluded | Not Ethiopian study | Published |  |
| 39 | Primožič et al [39] | Excluded | Not Ethiopian study | Published |  |
| 40 | Mumu et al [40] | Excluded | Not Ethiopian study | Published |  |
| 41 | Beverly et al [41] | Excluded | Not Ethiopian study | Published |  |
| 42 | Orzech et.al [42] | Excluded | Not Ethiopian study | Published |  |
| 43 | Agidew E. et al [43] | Excluded | No clear outcome of interest related to this study | Published |  |
| 44 | Jannoo et al [44] | Excluded | Not Ethiopian study | Published |  |
| 45 | Duarte et al [45] | Excluded | The outcome of interest not much  Not African study | Published |  |
| 46 | El–Abbassy, Amal [46] | Excluded | Not Ethiopian study | Published |  |
| 47 | Martinez-Harvell, Greisy [47] | Excluded | Not Ethiopian study | Published |  |
| 48 | Advika et al [48] | Excluded | Not Ethiopian study | Published |  |
| 49 | Mehrtak et al [49] | Excluded | Not Ethiopian study | Published |  |
| 50 | Chlebowy et al. [50] | Excluded | Not Ethiopian study | Published |  |
| 51 | Sorato, M. M et al [51] | Excluded | Not Ethiopian study | Published |  |
| 52 | MacDonald et al [52] | Excluded | Not Ethiopian study | Published |  |
| 53 | Kim, Hee-Seung et al. [53] | Excluded | Not Ethiopian study and The tool was not clearly utilize | Published |  |
| 54 | Taha, NADIA M et.al [54] | Excluded | Not Ethiopian study | Published |  |
| 55 | Bourne et al [55] | Excluded | Not Ethiopian study The tool was not clearly utilize | Published |  |
| 56 | Ezzat et al [56] | Excluded | Not Ethiopian study and The tool was not clearly utilize | Published |  |
| 57 | Salam et al. [57] | Excluded | Not African study and The tool was not clearly utilize | Published |  |
| 58 | Adam et al [58] | Excluded | Not Ethiopian study | Published |  |
| 59 | Owolabi et al [59] | Excluded | Not Ethiopian study | Published |  |
| 60 | Kurnia et al [60] | Excluded | Not Ethiopian study | Published |  |
| 61 | ODivya et al [61] | Excluded | Target Population difference | Published |  |
| 62 | Jarvie et al [62] | Excluded | Not Ethiopian study | Published |  |
| 63 | Tovar, Elizabeth Gressle et al [63] | Excluded | Not African study  The outcome of interest not clearly reported | Published |  |
| 64 | Bullard et al [64] | Excluded | Not Ethiopian study | Published |  |
| 65 | Lui et al [65] | Excluded | Not Ethiopian study | Published |  |
| 66 | MacPherson et al [66] | Excluded | Not African study | Published |  |
| 67 | Cox, Emily R et al [67] | Excluded | Not Ethiopian study | Published |  |
| 68 | Sumlin et al [68] | Excluded | Target Population difference | Published |  |
| 69 | Ferguson, Sarah et al. [69] | Excluded | No clear outcome of interest | Published |  |
| 70 | Mogre, Victor eta l [70] | Excluded | Reports not retrieved | Published |  |
| 71 | Phillips et al [71] | Excluded | Reports not retrieved | Published |  |
| 72 | Chen et al [72] | Excluded | Reports not retrieved | Published |  |
| 73 | Basu et al [73] | Excluded | Reports not retrieved | Published |  |
| 74 | Degefa et al [74] | Excluded | no clear outcome in line with this study | Published |  |
| 75 | Sharma et al [75] | Excluded | Not Ethiopian study | Published |  |
| 76 | Wang et al [76] | Excluded | Not Ethiopian study | Published |  |
| 77 | Gardner et al [77] | Excluded | Not Ethiopian study | Published |  |
| 78 | Marios, Tracy et al [78] | Excluded | Not Ethiopian study | Published |  |
| 79 | Simegn, Wudneh[79] | Excluded | no clear outcome in line with this study | Published |  |
| 80 | Mogre et al [80] | Excluded | Not Ethiopian study | Published |  |
| 81 | MichaliszynET et al [81] | Excluded | Not Ethiopian study | Published |  |
| 82 | Bonger z.et al. [82] | Excluded | No clear outcome of interest related to this study | Published |  |
| 83 | Oluma, Adugnaet al [83] | Excluded | No clear outcome of interest related to this study | Published |  |
| 84 | da Rocha, Rebeca B et.al [84] | Excluded | No clear outcome of interest related to this study | Published |  |
| 85 | Wondm, S. et al [85] | Excluded | No clear outcome of interest related to this study | Published |  |
| 86 | Shimels T . et al [86] | Excluded | No clear outcome of interest related to this study | Published |  |
| 87 | Tewahido, D. et al [87] | Excluded | No clear outcome of interest related to this study | Published |  |
| 88 | Mohammed M. et al [88] | Excluded | No clear outcome of interest related to this study | Published |  |

1. Debalke, R., B. Zinab, and T. Belachew, *Non adherence to physical activity recommendations and associated factors among Type 2 Diabetic patients in Illubabor zone, South West Ethiopia.* Glob J Obes Diabetes Metab Syndr, 2022. **9**(1): p. 001-006.

2. Zenu, S., et al., *Non-adherence to the World Health Organization’s physical activity recommendations and associated factors among healthy adults in urban centers of Southwest Ethiopia.* PLOS Global Public Health, 2023. **3**(1): p. e0001451.

3. Abate, H.K., Y.M. Ferede, and C.K. Mekonnen, *Adherence to physical exercise recommendations among type 2 diabetes patients during the COVID-19 pandemic.* International Journal of Africa Nursing Sciences, 2022. **16**: p. 100407.

4. Enyew, A., et al., *Prevalence and associated factors of physical inactivity among adult diabetes mellitus patients in Felege Hiwot Referral Hospital, Bahir Dar, Northwest Ethiopia.* Scientific Reports, 2023. **13**(1): p. 118.

5. Edmealem, A., S. Ademe, and B. Tegegne, *Level of physical activity and its associated factors among type II diabetes patients in dessie referral Hospital, northeast Ethiopia.* Diabetes, Metabolic Syndrome and Obesity: Targets and Therapy, 2020. **13**: p. 4067.

6. Zeleke Negera, G. and D. Charles Epiphanio, *Prevalence and predictors of nonadherence to diet and physical activity recommendations among type 2 diabetes patients in Southwest Ethiopia: a cross-sectional study.* International journal of endocrinology, 2020. **2020**.

7. Tamirat, A., L. Abebe, and G. Kirose, *Prediction of physical activity among Type-2 diabetes patients attending Jimma University specialized Hospital, southwest Ethiopia: Application of health belief model.* Science, 2014. **2**(6): p. 524-31.

8. Eshete, A., et al., *Effect of physical activity promotion program on adherence to physical exercise among patients with type II diabetes in North Shoa Zone Amhara region: a quasi-experimental study.* BMC public health, 2023. **23**(1): p. 709.

9. Koyra, H.C. and B.E. Doda, *Physical exercise and factors affecting among adult diabetic patients at Wolaita Soddo University teaching referral hospital, Southern Ethiopia.* International Journal of Physical Education, Sports and Health, 2017. **4**(5): p. 82-86.

10. Akumiah, P.O., et al., *Barriers to adherence to diet and exercise recommendation amongst type 2 diabetes mellitus patients.* J Health Med Nurs, 2017. **39**: p. 48-53.

11. Zhu, Y., et al., *Exercise adherence and compliance and its related factors among elderly patients with type 2 diabetes in china: a cross-sectional study.* Patient preference and adherence, 2022: p. 3329-3339.

12. Alhariri, A., et al., *Factors associated with adherence to diet and exercise among type 2 diabetes patients in Hodeidah city, Yemen.* Life, 2017. **7**(3): p. 264-271.

13. Ganiyu, A.B., et al., *Non-adherence to diet and exercise recommendations amongst patients with type 2 diabetes mellitus attending Extension II Clinic in Botswana.* African Journal of Primary Health Care and Family Medicine, 2013. **5**(1): p. 1-6.

14. Höchsmann, C., et al., *Effectiveness of a behavior change technique–based smartphone game to improve intrinsic motivation and physical activity adherence in patients with type 2 diabetes: randomized controlled trial.* JMIR serious games, 2019. **7**(1): p. e11444.

15. Saleh, F., et al., *Non-adherence to self-care practices & medication and health related quality of life among patients with type 2 diabetes: a cross-sectional study.* BMC public health, 2014. **14**: p. 1-8.

16. Parajuli, J., et al., *Factors associated with nonadherence to diet and physical activity among Nepalese type 2 diabetes patients; a cross sectional study.* BMC research notes, 2014. **7**: p. 1-9.

17. Ghimire, S., *Barriers to diet and exercise among Nepalese type 2 diabetic patients.* International Scholarly Research Notices, 2017. **2017**(1): p. 1273084.

18. Woodard, C.M. and M.J. Berry, *Enhancing adherence to prescribed exercise: structured behavioral interventions in clinical exercise programs.* Journal of Cardiopulmonary Rehabilitation and Prevention, 2001. **21**(4): p. 201-209.

19. Broadbent, E., L. Donkin, and J.C. Stroh, *Illness and treatment perceptions are associated with adherence to medications, diet, and exercise in diabetic patients.* Diabetes care, 2011. **34**(2): p. 338-340.

20. Forhan, M., et al., *Predicting exercise adherence for patients with obesity and diabetes referred to a cardiac rehabilitation and secondary prevention program.* Canadian journal of diabetes, 2013. **37**(3): p. 189-194.

21. Mkonka, L., et al., *Factors related to nonadherence to lifestyle modification in patients with diabetes mellitus type 2 at Harare Central Hospital.* IOSR Journal of Nursing and Health Science (IOSR-JNHS), 2016. **5**(5): p. 77-85.

22. Pandey, A., *Non-adherence to lifestyle (diet and exercise) modification recommendations among the type 2 diabetes mellitus patients in a tertiary level hospital.* J Inst Med, 2018. **41**(2).

23. Jadawala, H.D., et al., *Factors associated with non adherence to diet and physical activity among diabetes patients: a cross sectional study.* National Journal of Community Medicine, 2017. **8**(02): p. 68-73.

24. Joshi, R., D. Joshi, and P. Cheriyath, *Improving adherence and outcomes in diabetic patients.* Patient preference and adherence, 2017: p. 271-275.

25. Muhabuura, B., *Prevalence and factors associated with non-adherence to diet and exercise lifestyle recommendations among type 2 diabetic patients.* African journal of primary health care & family medicine, 2014. **4**(2): p. 110-120.

26. Darawad, M.W., et al., *Investigating physical exercise among Jordanians with diabetes mellitus.* Health, 2016. **8**(02): p. 181.

27. Wabe, N.T., M.T. Angamo, and S. Hussein, *Medication adherence in diabetes mellitus and self management practices among type-2 diabetics in Ethiopia.* North American journal of medical sciences, 2011. **3**(9): p. 418.

28. Abebe, A., et al., *Self-care practice and glycemic control among type 2 diabetes patients on follow up in a developing country: a prospective observational study.* Journal of Diabetes & Metabolic Disorders, 2022. **21**(1): p. 455-461.

29. Alhaiti, A.H., et al., *Adherence of type 2 diabetic patients to self‐care activity: Tertiary care setting in Saudi Arabia.* Journal of Diabetes Research, 2020. **2020**(1): p. 4817637.

30. Afaya, R.A., et al., *Medication adherence and self-care behaviours among patients with type 2 diabetes mellitus in Ghana.* PloS one, 2020. **15**(8): p. e0237710.

31. Dubasi, S.K., et al., *Questionnaire to assess adherence to diet and exercise advices for weight management in lifestyle-related diseases.* Journal of family medicine and primary care, 2019. **8**(2): p. 689-694.

32. Rustveld, L.O., et al., *Adherence to diabetes self-care behaviors in English-and Spanish-speaking Hispanic men.* Patient preference and adherence, 2009: p. 123-130.

33. Musenge, E.M., et al., *Glycaemic Control and Associated Self‐Management Behaviours in Diabetic Outpatients: A Hospital Based Observation Study in Lusaka, Zambia.* Journal of diabetes research, 2016. **2016**(1): p. 7934654.

34. Peng, X., et al., *A qualitative exploration of self-management behaviors and influencing factors in patients with type 2 diabetes.* Frontiers in endocrinology, 2022. **13**: p. 771293.

35. Nam, S., D.A. Dobrosielski, and K.J. Stewart, *Predictors of exercise intervention dropout in sedentary individuals with type 2 diabetes.* Journal of cardiopulmonary rehabilitation and prevention, 2012. **32**(6): p. 370-378.

36. Enikuomehin, A., et al., *Pattern of Self-care practices among type 2 diabetes patients in Southwest, Nigeria.* Nigerian journal of clinical practice, 2021. **24**(7): p. 978-985.

37. Muriungi, S.K. and D.M. Ndetei, *Effectiveness of psycho-education on depression, hopelessness, suicidality, anxiety and substance use among basic diploma students at Kenya Medical Training College.* South African Journal of Psychiatry, 2013. **19**(2): p. 41-50.

38. Makhubela, M., *Suicide and depression in university students: a possible epidemic*. 2021, SAGE Publications Sage UK: London, England. p. 3-5.

39. Primožič, S., et al., *Specific cognitive abilities are associated with diabetes self-management behavior among patients with type 2 diabetes.* Diabetes research and clinical practice, 2012. **95**(1): p. 48-54.

40. Mumu, S.J., et al., *Non-adherence to life-style modification and its factors among type 2 diabetic patients.* Indian journal of public health, 2014. **58**(1): p. 40-44.

41. Beverly, E.A. and L.A. Wray, *The role of collective efficacy in exercise adherence: a qualitative study of spousal support and type 2 diabetes management.* Health education research, 2010. **25**(2): p. 211-223.

42. Orzech, K.M., et al., *Diet and exercise adherence and practices among medically underserved patients with chronic disease: variation across four ethnic groups.* Health Education & Behavior, 2013. **40**(1): p. 56-66.

43. Agidew, E., et al., *Adherence to diabetes self-care management and associated factors among people with diabetes in Gamo Gofa Zone public health hospitals.* SAGE Open Medicine, 2021. **9**: p. 20503121211053953.

44. Jannoo, Z. and N.M. Khan, *Medication adherence and diabetes self-care activities among patients with type 2 diabetes mellitus.* Value in health regional issues, 2019. **18**: p. 30-35.

45. Duarte, C.K., et al., *Physical activity level and exercise in patients with diabetes mellitus.* Revista Da Associação Médica Brasileira (English Edition), 2012. **58**(2): p. 215-221.

46. El–Abbassy, A., *Non–adherence to lifestyle modification recommendations of Diet and Exercise amongst diabetic patients.* IOSR Journal of Nursing and Health Science (IOSR-JNHS)(Jul-Aug. 2015), 2015. **4**(4).

47. Martinez-Harvell, G., et al., *Predictors of adherence to physical activity guidelines in patients with diabetes mellitus in the US in 2017: An exploratory analysis.* Primary Care Diabetes, 2020. **14**(6): p. 645-653.

48. Advika, T., J. Idiculla, and S.J. Kumari, *Exercise in patients with Type 2 diabetes: Facilitators and barriers-A qualitative study.* Journal of family medicine and primary care, 2017. **6**(2): p. 288-292.

49. Mehrtak, M., A. Hemmati, and A. Bakhshzadeh, *Health Literacy and its Relationship with the medical, dietary Adherence and exercise in Patients with Type II Diabetes mellitus.* Journal of Health Literacy, 2018. **3**(2): p. 137-144.

50. Chlebowy, D.O., et al., *The relationships of demographic characteristics with diabetes biomarkers and physical activity adherence in African American adults.* Journal of Racial and Ethnic Health Disparities, 2016. **3**: p. 240-244.

51. Sorato, M.M., C. Tesfahun, and D. Lamessa, *Levels and predictors of adherence to self-care behaviour among adult type 2 diabetics at Arba Minch general hospital, Southern Ethiopia.* J Diabetes Metab, 2016. **7**(6): p. 11.

52. MacDonald, C.S., et al., *A systematic review of adherence to physical activity interventions in individuals with type 2 diabetes.* Diabetes/metabolism research and reviews, 2021. **37**(8): p. e3444.

53. Kim, H.-S., *A Survey of Blood Glucose Testing, Medication, Diet, and Exercise Adherence in Korean Patients with Type2 Diabetes.* Journal of Korean Academy of Fundamentals of Nursing, 2003. **10**(2): p. 181.

54. Taha, N.M., M. Abd El-Azeaz, and B.G. ABD EL-RAZIK, *Factors affecting compliance of diabetic patients toward therapeutic management.* Diabetes, 2005. **5**: p. 365-368.

55. Bourne, J.E., et al., *Brief exercise counseling and high-intensity interval training on physical activity adherence and cardiometabolic health in individuals at risk of type 2 diabetes: protocol for a randomized controlled trial.* JMIR research protocols, 2019. **8**(3): p. e11226.

56. Ezzat, A.M., et al., *The effects of interventions to increase exercise adherence in people with arthritis: a systematic review.* Musculoskeletal Care, 2015. **13**(1).

57. Salam, M.A. and A.F. Siddiqui, *Socio-demographic determinants of compliance among type 2 diabetic patients in Abha, Saudi Arabia.* Journal of clinical and diagnostic research: JCDR, 2013. **7**(12): p. 2810.

58. Adam, J. and L. Folds, *Depression, self-efficacy, and adherence in patients with type 2 diabetes.* The Journal for Nurse Practitioners, 2014. **10**(9): p. 646-652.

59. Owolabi, E.O., D. Ter Goon, and A.I. Ajayi, *Impact of mobile phone text messaging intervention on adherence among patients with diabetes in a rural setting: a randomized controlled trial.* Medicine, 2020. **99**(12): p. e18953.

60. Kurnia, I.D. and J.A. Rama, *The effect of theory of reasoned action implementation on dietary and physical activity adherence in patients with diabetes mellitus type 2.* Advances in Health Sciences Research, 2017. **3**(x): p. 233-236.

61. Divya, S. and P. Nadig, *Factors contributing to non-adherence to medication among type 2 diabetes mellitus in patients attending tertiary care hospital in South India.* Asian J Pharm Clin Res, 2015. **8**(2): p. 274-276.

62. Jarvie, J.L., et al., *Aerobic fitness and adherence to guideline-recommended minimum physical activity among ambulatory patients with type 2 diabetes mellitus.* Diabetes Care, 2019. **42**(7): p. 1333-1339.

63. Tovar, E.G., *Relationships between psychosocial factors and adherence to diet and exercise in adults with type 2 diabetes: A test of a theoretical model*. 2007.

64. Bullard, T., et al., *A systematic review and meta-analysis of adherence to physical activity interventions among three chronic conditions: cancer, cardiovascular disease, and diabetes.* BMC public health, 2019. **19**: p. 1-11.

65. Lui, K.C. and S.S. Hui, *Participation in and adherence to physical activity in people with physical disability.* Hong Kong Physiotherapy Journal, 2009. **27**(1): p. 30-38.

66. MacPherson, M.M., et al., *Using the behavior change wheel to develop text messages to promote diet and physical activity adherence following a diabetes prevention program.* Translational Behavioral Medicine, 2021. **11**(8): p. 1585-1595.

67. Cox, E.R., et al., *Potential utility of self-report measures of affect to optimise exercise adherence in people with type 2 diabetes.* Current Diabetes Reviews, 2019. **15**(4): p. 302-308.

68. Sumlin, L.L., et al., *Depression and adherence to lifestyle changes in type 2 diabetes: a systematic review.* The Diabetes Educator, 2014. **40**(6): p. 731-744.

69. Ferguson, S.S., *Exercise adherence in persons with type 2 diabetes and relationship to diabetes control*. 1998: University of Wisconsin--Madison.

70. Mogre, V., et al., *Adherence to and factors associated with self-care behaviours in type 2 diabetes patients in Ghana.* BMC Endocrine Disorders, 2017. **17**: p. 1-8.

71. Phillips, L.A., et al., *Self-management of chronic illness: the role of ‘habit’versus reflective factors in exercise and medication adherence.* Journal of Behavioral Medicine, 2016. **39**: p. 1076-1091.

72. Chen, D., et al., *Development of a behavior change intervention to improve physical activity adherence in individuals with metabolic syndrome using the behavior change wheel.* BMC Public Health, 2022. **22**(1): p. 1740.

73. Basu, S., et al., *Adherence to self-care practices, glycemic status and influencing factors in diabetes patients in a tertiary care hospital in Delhi.* World journal of diabetes, 2018. **9**(5): p. 72.

74. Degefa, G., et al., *Predictors of adherence toward specific domains of diabetic self-care among type-2 diabetes patients.* Clinical Medicine Insights: Endocrinology and Diabetes, 2020. **13**: p. 1179551420981909.

75. Sharma, A., et al., *Utilizing mobile technologies to improve physical activity and medication adherence in patients with heart failure and diabetes mellitus: Rationale and design of the TARGET-HF-DM Trial.* American heart journal, 2019. **211**: p. 22-33.

76. Wang, X., et al., *Validation of an information–motivation–behavioral skills model of upper limb functional exercise adherence among Chinese postoperative patients with breast cancer.* Breast Cancer, 2019. **26**: p. 198-205.

77. Gardner, A.W., et al., *Diabetic women are poor responders to exercise rehabilitation in the treatment of claudication.* Journal of vascular surgery, 2014. **59**(4): p. 1036-1043.

78. Marios, T., N.A. Smart, and S. Dalton, *The effect of tele-monitoring on exercise training adherence, functional capacity, quality of life and glycemic control in patients with type II diabetes.* Journal of sports science & medicine, 2012. **11**(1): p. 51.

79. Simegn, W., S.A. Mohammed, and G. Moges, *Adherence to Self-Care Practice Among Type 2 Diabetes Mellitus Patients Using the Theory of Planned Behavior and Health Belief Model at Comprehensive Specialized Hospitals of Amhara Region, Ethiopia: Mixed Method.* Patient preference and adherence, 2023: p. 3367-3389.

80. Mogre, V., et al., *A systematic review of adherence to diabetes self‐care behaviours: Evidence from low‐and middle‐income countries.* Journal of advanced nursing, 2019. **75**(12): p. 3374-3389.

81. Michaliszyn, S.F., M. Higgins, and M.S. Faulkner, *Patterns of physical activity adherence by adolescents with diabetes or obesity enrolled in a personalized community-based intervention.* The Diabetes Educator, 2018. **44**(6): p. 519-530.

82. Bonger, Z., S. Shiferaw, and E.Z. Tariku, *Adherence to diabetic self-care practices and its associated factors among patients with type 2 diabetes in Addis Ababa, Ethiopia.* Patient preference and adherence, 2018: p. 963-970.

83. Oluma, A., et al., *Predictors of adherence to self-care behavior among patients with diabetes at public hospitals in West Ethiopia.* Diabetes, Metabolic Syndrome and Obesity, 2020: p. 3277-3288.

84. da Rocha, R.B., C.S. Silva, and V.S. Cardoso, *Self-care in adults with type 2 diabetes mellitus: A systematic review.* Current diabetes reviews, 2020. **16**(6): p. 598-607.

85. Wondm, S.A., et al., *Association between self-care activities and glycemic control among patients with type 2 diabetes mellitus in Northwest Ethiopia general hospitals: a multicenter cross-sectional study.* Scientific Reports, 2024. **14**(1): p. 23198.

86. Shimels, T., M. Abebaw, and G.B. Gebretekle, *Poor Adherence to Common Recommendations and Associated Factors among Outpatients with Type 2 Diabetes Mellitus in a Police Hospital of Ethiopia.* Journal of Social Health and Diabetes, 2021. **9**(01): p. e8-e14.

87. Tewahido, D. and Y. Berhane, *Self-care practices among diabetes patients in Addis Ababa: a qualitative study.* PloS one, 2017. **12**(1): p. e0169062.

88. Mohammed, M.A. and N.T. Sharew, *Adherence to dietary recommendation and associated factors among diabetic patients in Ethiopian teaching hospitals.* Pan African Medical Journal, 2019. **33**(1).
