# Supplementary material for: A systematic review and meta-analysis of physical exercise non-adherence and its determinants among type 2 diabetic patients in Ethiopia
Source: PLoS One. 2024 Dec 4;19(12):e0314389. doi: 10.1371/journal.pone.0314389 (PMC11616846; doi:10.1371/journal.pone.0314389)
Supplement: Attachment — Submitted filename: Review comments_Plos One.docx [file pone.0314389.s008.docx]

The article written in a comprehensive manner and justifies the title. However, we came across several errors that needs to be corrected. In abstract, please specify the type of diabetic in introduction first line “type diabetic patients”. In methods, replace the word systematic review to meta-analysis in the sentence “Statistical analysis was performed using STATA version 14 and a **systemic review** was carried out using a random effect model method”.

Correct the date format - **June 5/ 2023 to June 29/ 6/2023** and **January 2013 to June 29/2023**. Complete the sentence “Any sort of disagreement between the research team while including and excluding articles on predefined criteria”

Please specify, data was extracted by two reviewers or four reviewers “Data were independently extracted by **four authors** using a standardized data extraction format of JBI as developed according to the 2014 Joanna Briggs Institute Reviewers’ Manual [26]. The tool includes Authors, study year, study design, sample size, prevalence, and risk of bias assessment score were included in the extraction. **The data were extracted by two independent reviewers** and any inconsistent data was cross-checked (supplementary file: Table S2).”

Please correct 113 articles are **not being ineligible** or not being eligible “the other 231 and 113 were removed for **not being ineligible** (study design and title difference) by automation tools and other reasons respectively.”

Correct the spelling of “among typ1 diabetes”, “asses”, “suplimentary table” and “metedological data quality”

Please recheck the number of excluded studies 74-51 is 23 not 22. Methodological quality assessment would come after the description of studies. Maintain the consistency of terminology throughout the article.

Correct the sentence in conclusion “as compared to most of the previous studies conducted else were in the world”. Author’s contribution should be in proper format. Correct the referencing format in reference no. 4 and 5.

Overall, major revisions are recommended.
