# Supplementary material for: A systematic review and meta-analysis of physical exercise non-adherence and its determinants among type 2 diabetic patients in Ethiopia
Source: PLoS One. 2024 Dec 4;19(12):e0314389. doi: 10.1371/journal.pone.0314389 (PMC11616846; doi:10.1371/journal.pone.0314389)
Supplement: Attachment — Submitted filename: PONE comment response final.docx [file pone.0314389.s009.docx]

**Authors’ point-by-point Response letter to the editor and Reviewers’ Reports**

**A systematic review and meta-analysis of physical exercise non-adherence and its determinants among type 2 diabetic patients in Ethiopia.**

**Authors:**

Hailemicahel Kindie Abate^1*^, Abere Woretaw Azage^1^, Muluken Chanie Agimas^2^, Gashaw Adane Nega^3^, Chilot Kassa Mekonnen^1^

**Authors’ response to Editor’s and Reviewers’ reports**

In the first place, we authors would like to give our heartfelt gratitude to Journal of “**plos one”** Editorial Team and the respective reviewers for reviewing our revised manuscript and providing the necessary comments that going to be corrected accordingly. As per the editors and reviewers’ comments, the authors tried to make corrections accordingly. This is a point-by-point letter to editors, reviewer 1, 2, and 3 reports.

**Note: the indicated page and line numbers are referred to the clean version of the manuscript**

PONE-D-23-20714

A systematic review and meta-analysis of physical exercise non-adherence and its determinants among type 2 diabetic patients in Ethiopia:

PLOS ONE

Dear Dr. Abate,

Thank you for submitting your manuscript to PLOS ONE. After careful consideration, we feel that it has merit but does not fully meet PLOS ONE’s publication criteria as it currently stands. Therefore, we invite you to submit a revised version of the manuscript that addresses the points raised during the review process.

==============================
**Dear Authors,**

**please see the comments made by the reviewers. There are some crucial points that need to be improve as recommended for systematic review and meta-analysis manuscripts.**

==============================

Please submit your revised manuscript by Apr 11 2024 11:59PM. If you will need more time than this to complete your revisions, please reply to this message or contact the journal office at [plosone@plos.org](mailto:plosone@plos.org). When you're ready to submit your revision, log on to <https://www.editorialmanager.com/pone/> and select the 'Submissions Needing Revision' folder to locate your manuscript file.

Please include the following items when submitting your revised manuscript:

- A rebuttal letter that responds to each point raised by the academic editor and reviewer(s). You should upload this letter as a separate file labeled 'Response to Reviewers'.

Authors’ response: Thank you Editor, we had submitted the disproof letter responded to each letter on comments given below.

- A marked-up copy of your manuscript that highlights changes made to the original version. You should upload this as a separate file labeled 'Revised Manuscript with Track Changes'.

Authors’ response: Thank you Editor, and we had submitted highlighted copy of original version one.

- An unmarked version of your revised paper without tracked changes. You should upload this as a separate file labeled 'Manuscript'.

Authors’ response: We had labeled and submitted accordingly.

If you would like to make changes to your financial disclosure, please include your updated statement in your cover letter. Guidelines for resubmitting your figure files are available below the reviewer comments at the end of this letter.

If applicable, we recommend that you deposit your laboratory protocols in [protocols.io](http://protocols.io/) to enhance the reproducibility of your results. Protocols.io assigns your protocol its own identifier (DOI) so that it can be cited independently in the future. For instructions see: <https://journals.plos.org/plosone/s/submission-guidelines#loc-laboratory-protocols>. Additionally, PLOS ONE offers an option for publishing peer-reviewed Lab Protocol articles, which describe protocols hosted on [protocols.io](http://protocols.io/). Read more information on sharing protocols at <https://plos.org/protocols?utm_medium=editorial-email&utm_source=authorletters&utm_campaign=protocols>.

We look forward to receiving your revised manuscript.

Kind regards,

Thiago Gomes Heck, Ph.D.
Academic Editor
PLOS ONE

Journal Requirements:

When submitting your revision, we need you to address these additional requirements.

1. Please ensure that your manuscript meets PLOS ONE's style requirements, including those for file naming. The PLOS ONE style templates can be found at 
<https://journals.plos.org/plosone/s/file?id=wjVg/PLOSOne_formatting_sample_main_body.pdf> and 
<https://journals.plos.org/plosone/s/file?id=ba62/PLOSOne_formatting_sample_title_authors_affiliations.pdf>

**Authors Response**: dear editor thank you for you constructive we had prepared as per the journal requirement .

2. Note from Emily Chenette, Editor in Chief of PLOS ONE, and Iain Hrynaszkiewicz, Director of Open Research Solutions at PLOS: Did you know that depositing data in a repository is associated with up to a 25% citation advantage (<https://doi.org/10.1371/journal.pone.0230416>)? If you’ve not already done so, consider depositing your raw data in a repository to ensure your work is read, appreciated and cited by the largest possible audience. You’ll also earn an Accessible Data icon on your published paper if you deposit your data in any participating repository (<https://plos.org/open-science/open-data/#accessible-data>).

**Authors Response:** Thank you we will make with reasonable request.

3. We noticed you have some minor occurrence of overlapping text with the following previous publication(s), which needs to be addressed:

- <https://www.sciencedirect.com/science/article/pii/S2214139122000142?via%3Dihub>
- <https://www.dovepress.com/getfile.php?fileID=68447>

In your revision ensure you cite all your sources (including your own works), and quote or rephrase any duplicated text outside the methods section. Further consideration is dependent on these concerns being addressed.

**Authors Response:** We had paraphrased. See : sections of other than the method.

4. We note that you have indicated that there are restrictions to data sharing for this study. PLOS only allows data to be available upon request if there are legal or ethical restrictions on sharing data publicly. For more information on unacceptable data access restrictions, please see <http://journals.plos.org/plosone/s/data-availability#loc-unacceptable-data-access-restrictions>.

Before we proceed with your manuscript, please address the following prompts:

a) If there are ethical or legal restrictions on sharing a de-identified data set, please explain them in detail (e.g., data contain potentially identifying or sensitive patient information, data are owned by a third-party organization, etc.) and who has imposed them (e.g., a Research Ethics Committee or Institutional Review Board, etc.). Please also provide contact information for a data access committee, ethics committee, or other institutional body to which data requests may be sent.

b) If there are no restrictions, please upload the minimal anonymized data set necessary to replicate your study findings to a stable, public repository and provide us with the relevant URLs, DOIs, or accession numbers. For a list of recommended repositories, please see
<https://journals.plos.org/plosone/s/recommended-repositories>. You also have the option of uploading the data as Supporting Information files, but we would recommend depositing data directly to a data repository if possible.

We will update your Data Availability statement on your behalf to reflect the information you provide.

**Authors Response:** We have submitted the data as supplementary file: See: page 16 line… 393

5. Please amend either the abstract on the online submission form (via Edit Submission) or the abstract in the manuscript so that they are identical.

Authors Response: thank you, we had made similar both in online and in the manuscript

6. Please include a separate caption for each figure in your manuscript.

Authors Response: Thank you we had made a separate caption for each figure in the manuscript. **See: page 17, line,401-420**

7. Please include a copy of Table 1 and 2 which you refer to in your text on page 11.

Authors Response: Thank you editor we had made correction in as per your comment. We had copied the tables under the text. See: page 10-12,

8. Please include captions for your Supporting Information files at the end of your manuscript, and update any in-text citations to match accordingly. Please see our Supporting Information guidelines for more information: <http://journals.plos.org/plosone/s/supporting-information>.

Authors Response: we had included the Supporting Information as per your comment. **See page 17…. Line 421-426**

Additional Editor Comments:

The manuscript is interesting and can found specialized audience. However, some fragilities in description and interpretation of the results need to be repair before acceptance.
Please, see the carefull comments made by the reviewers to improve some parts of the manuscript.

Authors Response: thank you, we try to modify the all points of the reviewers.

[Note: HTML markup is below. Please do not edit.]

Reviewers' comments:

Reviewer's Responses to Questions

**Comments to the Author**

1. Is the manuscript technically sound, and do the data support the conclusions?

The manuscript must describe a technically sound piece of scientific research with data that supports the conclusions. Experiments must have been conducted rigorously, with appropriate controls, replication, and sample sizes. The conclusions must be drawn appropriately based on the data presented.

Reviewer #1: Yes

Reviewer #2: Yes

Reviewer #3: Partly

Authors Response: Thank reviewer 1 and 2, and we try to addressed the reviewer 3 concern on the specific comment, given below.

2. Has the statistical analysis been performed appropriately and rigorously?

Reviewer #1: Yes

Reviewer #2: Yes

Reviewer #3: No

Authors Response: Thank reviewer 1 and 2, and we try to addressed the reviewer 3 concern on the specific comment, given below.

3. Have the authors made all data underlying the findings in their manuscript fully available?

The [PLOS Data policy](http://www.plosone.org/static/policies.action#sharing) requires authors to make all data underlying the findings described in their manuscript fully available without restriction, with rare exception (please refer to the Data Availability Statement in the manuscript PDF file). The data should be provided as part of the manuscript or its supporting information, or deposited to a public repository. For example, in addition to summary statistics, the data points behind means, medians and variance measures should be available. If there are restrictions on publicly sharing data—e.g. participant privacy or use of data from a third party—those must be specified.

Reviewer #1: Yes

Reviewer #2: Yes

Reviewer #3: No

Authors Response: Thank reviewer 1 and 2, and we try to addressed the reviewer 3 concern on the specific comment, given below.

4. Is the manuscript presented in an intelligible fashion and written in standard English?

PLOS ONE does not copyedit accepted manuscripts, so the language in submitted articles must be clear, correct, and unambiguous. Any typographical or grammatical errors should be corrected at revision, so please note any specific errors here.

Reviewer #1: No

Reviewer #2: Yes

Reviewer #3: Yes

Authors Response: Thank reviewer 2 and 3, and we try to address the reviewer 1concern on the specific comment, given below.

5. Review Comments to the Author

Please use the space provided to explain your answers to the questions above. You may also include additional comments for the author, including concerns about dual publication, research ethics, or publication ethics. (Please upload your review as an attachment if it exceeds 20,000 characters)

Authors Response: Thank you we had seen all the concerns of authors.

Reviewer #1: The article written in a comprehensive manner and justifies the title. However, we came across several errors that needs to be corrected. In abstract, please specify the type of diabetic in introduction first line “type diabetic patients”.

Authors Response: thank you we had mad correction as “type two diabetic patients” see page 2… line.. 28..

In methods, replace the word systematic review to meta-analysis in the sentence “Statistical analysis was performed using STATA version 14 and a systemic review was carried out using a random effect model method”.

Authors Response: thank you we had mad correction as “meta-analysis” see page 2… line.. 35..
Correct the date format - June 5/ 2023 to June 29/ 6/2023 and January 2013 to June 29/2023.

**Authors Response**: we had made correction to the format “January 1/ 2013 to June 29/2023” **See: .page 6, line 135-138**

Complete the sentence “Any sort of disagreement between the research team while including and excluding articles on predefined criteria”

Authors Response: Thank you we had made correction and complete the sentence as, “Any sort of disagreement between the research team while including and excluding articles could be solved by discussion based on the predefined criteria”. **Page 7, line, 178-181**

Please specify, data was extracted by two reviewers or four reviewers “Data were independently extracted by four authors using a standardized data extraction format of JBI as developed according to the 2014 Joanna Briggs Institute Reviewers’ Manual [26]. The tool includes Authors, study year, study design, sample size, prevalence, and risk of bias assessment score were included in the extraction. The data were extracted by two independent reviewers and any inconsistent data was cross-checked (supplementary file: Table S2).”

Authors Response: thank reviewers for the inconsistency the data were actually extracted by two reviewers. We had made correction on this typing error. **See: page 5 line 177, 183.**

Please correct 113 articles are not being ineligible or not being eligible “the other 231 and 113 were removed for not being ineligible (study design and title difference) by automation tools and other reasons respectively.”

Authors Response: thank you we had make correction on the wording problem.

**See: page 9, line 222**

Correct the spelling of “among typ1 diabetes”, “asses”, “suplimentary table” and “metedological data quality”

Authors Response: thank you we had make correction on the spelling, and we had revised the supplementary files. **See: page 9, line 235**

Please recheck the number of excluded studies 74-51 is 23 not 22.

Authors Response: thank you we had make correction on the numbering.

**See page: 9 line 226**

Methodological quality assessment would come after the description of studies.

Authors Response: thank you we had made as per your comment.

Maintain the consistency of terminology throughout the article.
Correct the sentence in conclusion “as compared to most of the previous studies conducted else were in the world”.

Authors Response: we had made correction on the sentence as “…. most of the previous conducted studies”.

Author’s contribution should be in proper format.

Authors Response: Thank you reviewer we had made correct ion on it.

Correct the referencing format in reference no. 4 and 5.

Authors Response: thank you we had made correction the format of reference 4and 5we had made correction on it.

Overall, major revisions are recommended.

Reviewer #2: The present study is a systematic review and meta-analysis on non-adherence to physical activity in patients diagnosed with type 2 diabetes mellitus in the Ethiopian population. The structure of the study is very well thought out in terms of conduction, data extraction and selection of included studies. A very positive point for the quality of the study was that the statistical tools for conducting meta-analysis, meta-regression and sensitivity analysis were present in the manuscript. The written language is adequate, although it presents some small spelling errors that do not significantly affect the overall quality of the manuscript. Despite these positive points and which demonstrate that the researchers were concerned with conducting the study in the best way possible, some pillars of the manuscript's structure worry me, as they may have failed to include studies in this systematic review. Please look carefully at my suggestions/questions/comments below:

Authors Response: thank you, we revised the overall comments given below

1. Do the authors have the project for this systematic review registered on the PROSPERO platform? The CRD approval number must be present on the abstract and in the methods section.

Authors Response: thank you reviewer we had made registration prior to the preparation of the manuscript. Sorry for missing in the write up. Now we add this number on the method section. **See page 5, line 115**

2. Please complete the checklist for the abstract in accordance with the PRISMA 2020 protocol and include it as a supplementary document to the manuscript.

Authors Response: we had already prepared and submitted as supplementary document in the system and put as a supplementary file at the end of the manuscript under the heading supplementary file. **Page: 17, line: 421**

3. The authors used EMBASE as a database, but only used terms indexed for MeSH. I strongly recommend that authors carry out a new search with terms indexed from the European platform that is specifically directed to EMBASE, Emtree.

Authors Response: thank you, we had used EMBASE, Emtree as you directed.

See: **Page 5, line 125-130**

4. Why did the authors not include gray literature as an additional strategy? It is described in the methodology very vaguely, but the selected articles are not present in Figure 1, whether they were preprints or even the gray literature bases used for this systematic review. For this systematic review design, I believe it is an interesting addition to broaden the range of researchers.

Authors Response: we try to search grey literatures using the searching engine such as Google advance and Google scholar but we had not found any grey literatures in this study title. **See: page 9 line, 233**

5. Please add the search strategy with the terms MeSH, Emtree with their indexed terms and their synonyms as a supplementary document. In this case, I suggest including search terms for observational studies (as was the focus of this manuscript). I even suggest that authors learn more about the studies used in this Cochrane review published in 2019, which evaluated precisely this issue for search engines.

Reference:
Li L, Smith HE, Atun R, Tudor Car L. Search strategies to identify observational studies in MEDLINE and Embase. Cochrane Database of Systematic Reviews 2019, Issue 3. Art. No.: MR000041. DOI: 10.1002/14651858.MR000041.pub2. Accessed 05 January 2024.
<https://www.cochranelibrary.com/cdsr/doi/10.1002/14651858.MR000041.pub2/full>

Authors Response: we had submitted the supplementary file as modeled by EMBASE Emtree and cochorian libray using title (Ti) and abstract (Ab). **See: page, 9 lines, 233**

6. I suggest that the authors carry out a new search with more diversified terms in the domain of adherence to physical exercise, as the terms used by the authors may bring fewer studies than the databases can have (my concern is related to the indexing of terms for the primary outcome established by the authors for this systematic review). To do this, I suggest reading supplementary Table 1 of the article cited below as inspiration:

Reference:
El Haddad L, Peiris CL, Taylor NF, McLean S. Determinants of Non-Adherence to Exercise or Physical Activity in People with Metabolic Syndrome: A Mixed Methods Review. Patient Prefer Adherence. 2023 Feb 3;17:311-329. doi: 10.2147/PPA.S383482. PMID: 36760232; PMCID: PMC9904214.

Authors Response: We try to use the adherence to physical exercise; still there were no new studies on the predefined period of data extraction. **See: page 5 line 126**

Reviewer #3: Dear Editors and Authors.
Thank you for giving me the opportunity to get to know your work and to collaborate with you.
Please find below what I have contributed.

The study aims to assess the prevalence and reasons for non-adherence to physical activity in people with DM2.
The introduction is appropriate but, although justified, it seems too focused on the country in question. This may limit the external validity of the study.

Authors Response: Thank you we try oversee the trends of exercise none-adherence of diabetic patient widely. **See: page 3, line 61 -66**

In the methodology, the authors clearly describe the research strategies, the database, the tools used to assess the risk of bias in the studies, and the statistical methods used to analyze the data.
The presentation of the results is partially satisfactory (forest plot) and the discussion is in line with the use of the available data. The conclusion is partially consistent with the objectives of the study and the data found.
However, in my opinion, there are some points that need clarification, which, if considered by the authors, could contribute to the quality of the work.

Authors Response: thank you we try to made correction based your detail comment below.

Introduction
The introduction presents the research question well. However, although it is justified, it seems to me to be extremely local.
Authors Response: Thank you we try oversee the trends of exercise none-adherence of diabetic patient widely. **See: page 3, line 61 -66**

Methods
Inform PROSPERO registration number.
Authors Response: thank you reviewer we had made registration prior to the preparation of the manuscript. Sorry for missing in the write up. Now we add this number on the method section. **See: page 5 line 115**

Searching strategies
Change “PubMed” to “Medline/PubMed”

Authors Response: Thank you we had changed as per your comment. **See. page 5 line 125**

Explain the reason for limiting the date range of the search. I see no reason to limit the search to the last 10 years.

Authors Response: This just not for limitation the “…..year articles published from January 1/ 2013 to June 29/2023 was included” mean that the first year “January 1/ 2013 indicates there is no study before which related with exercise none adherence of type two diabetic patients and the last year June 29/2023 mean that the last day of the searching of articles for this manuscript. **See: page 6 line 136-139**

Was grey literature searched? If so, where?

Authors Response: we try to search unpublished reports (thesis, dissertations) using Google and Google scholar but we couldn’t obtain any grey literature in line with the study title. **See, page 5, line 118**

Eligibility criteria
Study Inclusion and Exclusion criteria.
Poor methodological quality is not a criterion for excluding studies. These studies must be included and analyzed. If necessary, a subgroup analysis can be performed.

Authors Response: thank you reviewer this is problem write up it is not due methodological quality rather studies due to the low quality appraisal score were excluded in the analysis of the study. **Page 7, Line 160-170**

I suggest including in the methods the reason for including in the subgroup analysis only the variables of sex, place of residence and level of education.

Authors Response: Thank you reviewers, we try to select many other variables for analysis but there was inconsistency across the finding the result of the included studies.

I recommend using GRADE to assess the quality of evidence generated by meta-analysis.
Authors Response: We had already GRADE and submitted as supplementary file.

See page line. **Page 17: line 426**

Results
Page 10, line 5 - …”those articles conducted among typ1 diabetes” * please, correct “type”.
Authors Response: thank you we had make correction on it.
I suggest that a table similar to Table 1 in the PRISMA checklist be included in the paper.

Authors Response: we had submitted as supplementary file. **See page 17, line 424**

In addition to the data in this table, I propose to include the variables analyzed, sex, place of residence and level of education.

Authors Response: thank you we had fill all necessary information using the PRISMA checklist table. **See page 17, line 424**

Assessment of heterogeneity – page 11
“The analysis result showed that the source of heterogeneity is not due to region (p=0.0001 I2 =100).” I don’t understand this result. p<0,05 and I2 100% isn’t an indicator of heterogeneity? If I have understood this result correctly, it means that there is a high degree of heterogeneity (The authors indicated this condition when describing the analysis of heterogeneity in the methods). Furthermore, the difference between the states/regions, suggests this difference.

Authors Response: dear reviewer thank you for your intensive comment and sorry for my misinterpretation. The result the above heterogeneity test of sub-group analysis based on region showed that there was significant heterogeneity due to region. Therefore, it was corrected accordingly

**See: page 11, line 268**

The pooled analysis of determinant factors - Page 12

The effect of the sex of participants

Heterogeneity and Publication bias of the included studies
“As stated figure 6 showed that the overall heterogeneity test (I2) on the effect of being female sex was 0.0% with a p-value of 0.977, using a random effect model to adjust the observed variability. This heterogeneity test indicates there is observed variability across the included studies.”
Again, in my opinion, the authors have confused the results of the heterogeneity test.

Authors Response: dear reviewer thank you for your intensive comment and sorry for my misinterpretation. The result the above heterogeneity test of the included studies indicated that there is no observed variability across the included studies. Therefore, it was corrected accordingly.

**See: page 12 line 293**

The effect of primary education
Heterogeneity and Publication bias of the included studies – page 13
“As stated above in Figure 8, the overall heterogeneity test (I2) on the effect of primary education was 0.0% with a p-value of 0.548, using a random effect model to adjust the observed variability. This heterogeneity test indicates there is observed variability across the included studies.”
Same comment as above.

**Authors Response**: Again dear reviewer thank you for your intensive comment and sorry for my misinterpretation. The result the above heterogeneity test of the included studies indicated that there is no observed variability across the included studies. Therefore, it was corrected accordingly. **See: page 13	line, 304**

The effect of rural residency
Heterogeneity and Publication bias of the included studies
“As stated above in Figure 10, the overall heterogeneity test (I2) on the effect of being in rural residency was 20.2% with a p-value of 0.057, using a random effect model to adjust the observed variability. This heterogeneity test indicates there is observed variability across the included studies.”
Same comment as above.

Authors Response: in in the same way sorry for my misinterpretation. The result the above heterogeneity test of the included studies indicated that there is mild observed variability across the included studies. Therefore, it was corrected accordingly. .

**See: page 13	line, 315**

Discussion

My suggestion is to review the results of the heterogeneity analysis and, if my analysis is correct, to explore the reasons for the heterogeneity in the discussion, as it is not possible to do a meta-regression due to the small number of studies included.

Authors Response: we review the heterogeneity test interpretations and correct accordingly and we removed the meta-regression.

In addition, I suggest that the individual quality of the studies and the quality of the evidence (GRADE) be examined in each of the topics of discussion.

Authors Response: We had assessed the quality of each topic of the tool and submitted as supplementary file.

**See: page 17, line, 426**

Regarding the limitations of the study, I suggest highlighting the small number of studies and states included, and, depending on the quality of the evidence, reporting this as a limitation of the study.

Authors Response: thank you reviewer we accept your suggestions. **See: page 15, line, 454-457**

Conclusion
In the conclusion of the study, my suggestion is to minimize the impact of the results due to the limitations that are presented.

Authors Response: Thank you reviewer we accept your suggestions, we try to minimize the limitation.

6. PLOS authors have the option to publish the peer review history of their article ([what does this mean?](https://journals.plos.org/plosone/s/editorial-and-peer-review-process#loc-peer-review-history)). If published, this will include your full peer review and any attached files.


If you choose “no”, your identity will remain anonymous but your review may still be made public.


**Do you want your identity to be public for this peer review?** For information about this choice, including consent withdrawal, please see our [Privacy Policy](https://www.plos.org/privacy-policy).

Reviewer #1: No

Reviewer #2: **Yes:** Giuseppe Potrick Stefani

Reviewer #3: **Yes:** LUIS FERNANDO DERESZ
