# Supplementary material for: A systematic review and meta-analysis of physical exercise non-adherence and its determinants among type 2 diabetic patients in Ethiopia
Source: PLoS One. 2024 Dec 4;19(12):e0314389. doi: 10.1371/journal.pone.0314389 (PMC11616846; doi:10.1371/journal.pone.0314389)
Supplement: Attachment — Submitted filename: Response to Reviwers.docx [file pone.0314389.s010.docx]

PONE-D-23-20714R1

A systematic review and meta-analysis of physical exercise non-adherence and its determinants among type 2 diabetic patients in Ethiopia:

PLOS ONE

Dear Dr. Abate,

Kind regards,

Thiago Gomes Heck, Ph.D.
Academic Editor
PLOS ONE

Journal Requirements:

Please review your reference list to ensure that it is complete and correct. If you have cited papers that have been retracted, please include the rationale for doing so in the manuscript text, or remove these references and replace them with relevant current references. Any changes to the reference list should be mentioned in the rebuttal letter that accompanies your revised manuscript. If you need to cite a retracted article, indicate the article’s retracted status in the References list and also include a citation and full reference for the retraction notice.

**Author Response**: All reference were checked and completed.

Additional Editor Comments:

Dear authors,
The manuscript is interestinlgy and can be accept after minor changes.
Please observe carefully the comments made by reviewer 2 below:

Dear Editor and Authors!
Thank you for the opportunity to review the paper again. In this analysis I can see the effort and progress of the work, but in my opinion, there are still adjustments to be made that could improve the quality of the study.

Abstract – Use the PRISMA for Abstracts Checklist – include PROSPERO registration number

**Author response**: we had used the PRISMA for Abstracts Checklist uploaded in the system and include the ROSPERO online database registration number of CRD42023430579 in method section

Methods
Searching strategies

Line 118 – Distinguish in the text between searches in traditional databases and searches in gray literature. In addition, Figure 1 needs to be modified to show searches and results from indexed databases and grey literature separately.

**Authors’ response**: we had distinguished the text searches in traditional databases and searches in gray literature and figure one is modified accordingly.

In addition, there are other databases besides Google for searching gray literature, [clinicaltrials.gov](http://clinicaltrials.gov/), International Clinical Trials Registry Platform (ICTRP), The European Union Clinical Trials, OpenGrey, ProQuest.

**Authors’ response**: we made used additional search engine like opengery, proQuest….

125 – Correct the word "Cochrane" wherever it appears.

**Authors’ response**: Thank you we had made correction on the word “Cochrane”

130 – S2 file - Present the complete search strategy used in each included database in the Supplementary Appendix.
**Authors’ response:** we had supplied complete searching strategies of the included database.

137 – Correct the word MedLine

**Authors’ response:** we had corrected it

149 – Correct the word PubMed

**Authors’ response**: we had corrected it
In the text, state that there were no year or language restrictions in the database search.

**Authors’ response:** Thank you, we had made correction as per your comment.

Eligibility criteria
Study Inclusion and Exclusion criteria

Lines 157 to 160
Quantitative studies that reported the prevalence of overall physical exercise non-adherence of type 2 diabetic patients, master's thesis, and dissertations were included in the study, whereas qualitative study design, single case study research reports, not fully accessed articles, and poor methodological quality were excluded from the analysis.

As suggest in the first revision: “Poor methodological quality were excluded from the analysis". - Poor methodological quality is not a criterion for excluding studies.
After the critical appraisal, the reviewers decided to include or exclude screened articles based on the overall quality of the appraisal score out of 9. The article was prone to exclude when the score was below average, which is of three independent reviewers.

Authors Response: thank you reviewer this is problem write up it is not due methodological quality rather studies due to the low quality appraisal score were excluded in the analysis of the study.

Review reply: Since no studies were excluded because of low quality (which is methodologically inappropriate), I suggest removing this sentence to avoid confusion.

Authors’ response: Thank you we had removed the text.
As suggest in the first revision: I recommend using GRADE to assess the quality of evidence generated by meta-analysis.
Authors Response: We had already GRADE and submitted as supplementary file.
See page line. Page 17: line 426
Review reply: GRADE is different to Newcastle-Ottawa Scale or JBI see the reference Guyatt, Gordon H., et al. "GRADE guidelines: a new series of articles in the Journal of Clinical Epidemiology." Journal of Clinical Epidemiology 64.4 (2011): 380-382.

Authors response: we had used the GRADE ON the method section to assess the included study.

Results
Line 246 – Correct typ”e” one diabetes

Authors response: We had made correction

Line 252 Description of included studies
“All articles were conducted with a cross-sectional study design with the smallest prevalence from Oromia (11.9%) [34] and the largest prevalence from Amhara 73.6% [35] regional states of Ethiopia. On the contrary, the largest sample size was from the Oromia (1191) [36], whereas the smallest sample size was from Amhara (302) regional states of Ethiopia [37].”
This point must be used in discussion.

**Author response**: thank you reviewer, this is just for description of the included study it is not the objective of the meta-analysis. We had clearly discussed the prevalence its determinants as the objective of the study.

Line 263 Quality assessment of included studies
The outcome of the quality appraisal ranged from moderate to high methodological quality, in which two studies scored 9 points [36, 39], three studies scored 8 [34, 35, 38], and the other two studies scored 7 268 [37] [40] (S3 file).

**Author response:** We had revised it, and we had removed the methodological quality score and changed quality score of the included study based on Newcastle-Ottawa Scale score Discussion

As pointed out above, this topic “All articles were conducted with a cross-sectional study design with the smallest prevalence from Oromia (11.9%) [34] and the largest prevalence from Amhara 73.6% [35] regional states of Ethiopia. On the contrary, the largest sample size was from the Oromia (1191) [36], whereas the smallest sample size was from Amhara (302) regional states of Ethiopia [37].” must be used in discussion. For example, the largest Amhara prevalence cannot be influenced by small sample size? In addition, the same point may be made in the study limitations.

**Author response**: Thank you, The above information is just description of the included study. The prevalence and the sample size of each study may not be inline since the participants from total study might be different. For example if a study participant is higher in Amhara region compared to Oromia the prevalence from Amhara region becomes higher. This can be checked from each study’s findings. Further, the objective of this study is to find and discussed the pooled prevalence exercise non adherence not the finding of the other researcher individual prevalence.

Again, I suggest that the quality of the evidence (GRADE) be examined in each of the topics of discussion and in the study limitations.

Author response: We had submitted GRADE quality of evidence in method and result section and limitation section of the study.

Conclusion
In the conclusion of the study, my suggestion is to minimize the impact of the results due to the limitations that are presented.

Author response: we had made revision on it.

[Note: HTML markup is below. Please do not edit.]

Reviewers' comments:

Reviewer's Responses to Questions

**Comments to the Author**

1. If the authors have adequately addressed your comments raised in a previous round of review and you feel that this manuscript is now acceptable for publication, you may indicate that here to bypass the “Comments to the Author” section, enter your conflict of interest statement in the “Confidential to Editor” section, and submit your "Accept" recommendation.

Reviewer #2: All comments have been addressed

Reviewer #3: (No Response)

Author: thank you reviewer 2 for your constructive comments

2. Is the manuscript technically sound, and do the data support the conclusions?

The manuscript must describe a technically sound piece of scientific research with data that supports the conclusions. Experiments must have been conducted rigorously, with appropriate controls, replication, and sample sizes. The conclusions must be drawn appropriately based on the data presented.

Reviewer #2: Yes

Reviewer #3: Partly

We had made revision for reviewer 3

3. Has the statistical analysis been performed appropriately and rigorously?

Reviewer #2: Yes

Reviewer #3: Yes

Author response thank you reviewers

4. Have the authors made all data underlying the findings in their manuscript fully available?

The [PLOS Data policy](http://www.plosone.org/static/policies.action#sharing) requires authors to make all data underlying the findings described in their manuscript fully available without restriction, with rare exception (please refer to the Data Availability Statement in the manuscript PDF file). The data should be provided as part of the manuscript or its supporting information, or deposited to a public repository. For example, in addition to summary statistics, the data points behind means, medians and variance measures should be available. If there are restrictions on publicly sharing data—e.g. participant privacy or use of data from a third party—those must be specified.

Reviewer #2: Yes

Reviewer #3: No

Author Response: thank you reviwers we had add Data Availability Statement and other requirement of the plos one journal

5. Is the manuscript presented in an intelligible fashion and written in standard English?

PLOS ONE does not copyedit accepted manuscripts, so the language in submitted articles must be clear, correct, and unambiguous. Any typographical or grammatical errors should be corrected at revision, so please note any specific errors here.

Reviewer #2: Yes

Reviewer #3: Yes

Author Response: thank you reviewers for your constructive comments

6. Review Comments to the Author

Please use the space provided to explain your answers to the questions above. You may also include additional comments for the author, including concerns about dual publication, research ethics, or publication ethics. (Please upload your review as an attachment if it exceeds 20,000 characters)

Reviewer #2: Thank you for you point-by-point response.
All comments and suggestions have been adequately addressed by the authors.

Reviewer #3: Dear Editor and Authors!
Thank you for the opportunity to review the paper again. In this analysis I can see the effort and progress of the work, but in my opinion, there are still adjustments to be made that could improve the quality of the study.

Abstract – Use the PRISMA for Abstracts Checklist – include PROSPERO registration number

**Author response**: we had used the PRISMA for Abstracts Checklist uploaded in the system and include the ROSPERO online database registration number of CRD42023430579 in method section

Methods
Searching strategies

Line 118 – Distinguish in the text between searches in traditional databases and searches in gray literature. In addition, Figure 1 needs to be modified to show searches and results from indexed databases and grey literature separately.

**Authors’ response**: we had distinguished the text searches in traditional databases and searches in gray literature and figure one is modified accordingly.


In addition, there are other databases besides Google for searching gray literature, [clinicaltrials.gov](http://clinicaltrials.gov/), International Clinical Trials Registry Platform (ICTRP), The European Union Clinical Trials, OpenGrey, ProQuest.

**Authors’ response**: we made used additional search engine like opengery, proQuest….

125 – Correct the word "Cochrane" wherever it appears.

**Authors’ response**: Thank you we had made correction on the word “Cochrane”

130 – S2 file - Present the complete search strategy used in each included database in the Supplementary Appendix.

**Authors’ response:** we had supplied complete searching strategies of the included database

137 – Correct the word MedLine
**Authors’ response**: we had corrected it

149 – Correct the word PubMed

**Authors’ response:** we had corrected it

In the text, state that there were no year or language restrictions in the database search.
**Authors’ response:** Thank you, we had made correction as per your comment

Eligibility criteria
Study Inclusion and Exclusion criteria

Lines 157 to 160
Quantitative studies that reported the prevalence of overall physical exercise non-adherence of type 2 diabetic patients, master's thesis, and dissertations were included in the study, whereas qualitative study design, single case study research reports, not fully accessed articles, and poor methodological quality were excluded from the analysis.

As suggest in the first revision: “Poor methodological quality were excluded from the analysis". - Poor methodological quality is not a criterion for excluding studies.
After the critical appraisal, the reviewers decided to include or exclude screened articles based on the overall quality of the appraisal score out of 9. The article was prone to exclude when the score was below average, which is of three independent reviewers.

Authors Response: thank you reviewer this is problem write up it is not due methodological quality rather studies due to the low quality appraisal score were excluded in the analysis of the study.

Review reply: Since no studies were excluded because of low quality (which is methodologically inappropriate), I suggest removing this sentence to avoid confusion.

Authors’ response: Thank you we had removed the text.

As suggest in the first revision: I recommend using GRADE to assess the quality of evidence generated by meta-analysis.
Authors Response: We had already GRADE and submitted as supplementary file.
See page line. Page 17: line 426
Review reply: GRADE is different to Newcastle-Ottawa Scale or JBI see the reference Guyatt, Gordon H., et al. "GRADE guidelines: a new series of articles in the Journal of Clinical Epidemiology." Journal of Clinical Epidemiology 64.4 (2011): 380-382.
Authors response: we had used the GRADE ON the method section to assess the included study.

Results
Line 246 – Correct typ”e” one diabetes

Authors response: We had made correction

Line 252 Description of included studies
“All articles were conducted with a cross-sectional study design with the smallest prevalence from Oromia (11.9%) [34] and the largest prevalence from Amhara 73.6% [35] regional states of Ethiopia. On the contrary, the largest sample size was from the Oromia (1191) [36], whereas the smallest sample size was from Amhara (302) regional states of Ethiopia [37].”
This point must be used in discussion.

Line 263 Quality assessment of included studies
The outcome of the quality appraisal ranged from moderate to high methodological quality, in which two studies scored 9 points [36, 39], three studies scored 8 [34, 35, 38], and the other two studies scored 7 268 [37] [40] (S3 file).
**Author response**: thank you reviewer, this is just for description of the included study it is not the objective of the meta-analysis. We had clearly discussed the prevalence its determinants as the objective of the study.


Discussion

As pointed out above, this topic “All articles were conducted with a cross-sectional study design with the smallest prevalence from Oromia (11.9%) [34] and the largest prevalence from Amhara 73.6% [35] regional states of Ethiopia. On the contrary, the largest sample size was from the Oromia (1191) [36], whereas the smallest sample size was from Amhara (302) regional states of Ethiopia [37].” must be used in discussion. For example, the largest Amhara prevalence cannot be influenced by small sample size? In addition, the same point may be made in the study limitations.

**Author response**: Thank you, The above information is just description of the included study. The prevalence and the sample size of each study may not be inline since the participants from total study might be different. For example if a study participant is higher in Amhara region compared to Oromia the prevalence from Amhara region becomes higher. This can be checked from each study’s findings. Further, the objective of this study is to find and discussed the pooled prevalence exercise non adherence not the finding of the other researcher individual prevalence.

Again, I suggest that the quality of the evidence (GRADE) be examined in each of the topics of discussion and in the study limitations.

Author response: We had submitted GRADE quality of evidence in method and result section and limitation section of the study .

Conclusion
In the conclusion of the study, my suggestion is to minimize the impact of the results due to the limitations that are presented.

Author response: we had made revision on it.

7. PLOS authors have the option to publish the peer review history of their article ([what does this mean?](https://journals.plos.org/plosone/s/editorial-and-peer-review-process#loc-peer-review-history)). If published, this will include your full peer review and any attached files.


If you choose “no”, your identity will remain anonymous but your review may still be made public.


**Do you want your identity to be public for this peer review?** For information about this choice, including consent withdrawal, please see our [Privacy Policy](https://www.plos.org/privacy-policy).

Reviewer #2: **Yes:** Giuseppe Potrick Stefani

Reviewer #3: **Yes:** Luís Fernando Deresz

[NOTE: If reviewer comments were submitted as an attachment file, they will be attached to this email and accessible via the submission site. Please log into your account, locate the manuscript record, and check for the action link "View Attachments". If this link does not appear, there are no attachment files.]

While revising your submission, please upload your figure files to the Preflight Analysis and Conversion Engine (PACE) digital diagnostic tool, <https://pacev2.apexcovantage.com/>. PACE helps ensure that figures meet PLOS requirements. To use PACE, you must first register as a user. Registration is free. Then, login and navigate to the UPLOAD tab, where you will find detailed instructions on how to use the tool. If you encounter any issues or have any questions when using PACE, please email PLOS at [figures@plos.org](mailto:figures@plos.org). Please note that Supporting Information files do not need this step.

*In compliance with data protection regulations, you may request that we remove your personal registration details at any time.* [*(Remove my information/details)*](https://www.editorialmanager.com/pone/login.asp?a=r)*. Please contact the publication office if you have any questions.*

|  | ReplyForward  You can't react to a message with a reply-to address |
| --- | --- |

Page 1 of 10
