# Supplementary material for: A systematic review and meta-analysis of physical exercise non-adherence and its determinants among type 2 diabetic patients in Ethiopia
Source: PLoS One. 2024 Dec 4;19(12):e0314389. doi: 10.1371/journal.pone.0314389 (PMC11616846; doi:10.1371/journal.pone.0314389)
Supplement: Attachment — Submitted filename: Response to reviwers.docx [file pone.0314389.s011.docx]

Editorial office comments and response

Please include the following items when submitting your revised manuscript:

- A rebuttal letter that responds to each point raised by the academic editor and reviewer(s). You should upload this letter as a separate file labeled 'Response to Reviewers'.
- A marked-up copy of your manuscript that highlights changes made to the original version. You should upload this as a separate file labeled 'Revised Manuscript with Track Changes'.
- An unmarked version of your revised paper without tracked changes. You should upload this as a separate file labeled 'Manuscript'.

If you would like to make changes to your financial disclosure, please include your updated statement in your cover letter. Guidelines for resubmitting your figure files are available below the reviewer comments at the end of this letter.

If applicable, we recommend that you deposit your laboratory protocols in protocols.io to enhance the reproducibility of your results. Protocols.io assigns your protocol its own identifier (DOI) so that it can be cited independently in the future. For instructions see: <https://journals.plos.org/plosone/s/submission-guidelines#loc-laboratory-protocols>. Additionally, PLOS ONE offers an option for publishing peer-reviewed Lab Protocol articles, which describe protocols hosted on protocols.io. Read more information on sharing protocols at <https://plos.org/protocols?utm_medium=editorial-email&utm_source=authorletters&utm_campaign=protocols>.

We look forward to receiving your revised manuscript.

Kind regards,

Philipp Baumert
Academic Editor
PLOS ONE

Journal Requirements:

Please review your reference list to ensure that it is complete and correct. If you have cited papers that have been retracted, please include the rationale for doing so in the manuscript text, or remove these references and replace them with relevant current references. Any changes to the reference list should be mentioned in the rebuttal letter that accompanies your revised manuscript. If you need to cite a retracted article, indicate the article’s retracted status in the References list and also include a citation and full reference for the retraction notice.

Author Response: All reference were checked and completed.

Additional Editor Comments:

Dear Hailemichael Kindie Abate and Co-authors,

Thank you for your thorough revision of the manuscript. I was recently assigned the role of academic editor and generally aim to minimise the need for repeated revisions. However, due to my later involvement in this process, I encourage you to carefully address the comments from Reviewer 2. With these adjustments, I am confident that your manuscript will be strengthened, and I hope we can finalise this process promptly.

To clarify a few points:
- Line 120: As noted by Reviewer 2, Google Scholar is not a traditional indexed database. Please include it under grey literature and add the missing comma as suggested.

**Author Response;** Thank you we had made correction as per your comment.

- Figure 1: Am I right that the search results for OpenGrey and ProQuest are missing in figure 1? Please kindly include these.

**Author Response:** Thank you, we had made correction on figure 1

- Lines 169–171: Reviewer 2 has suggested removing this sentence if no articles were removed based on quality checks. Please either provide an example of excluded articles in your revision or delete this sentence if none were removed.

**Author** Response: thank you we had cited the articles removed from the analysis based on quality checks

**See page 7 Line 178** and the supplementary file for quality checks

- - Lastly, in the conclusion (full text and Abstract), please consider adding a brief statement that the results should be interpreted with caution due to the low certainty of evidence. You may choose to reference the GRADE score in this context (or not).

Author Response: Whe had put the result with text and supplementary file. **See page 9 line 223-2232 page: 14 line 336-351** the detail on result section. We also stated on the abstract section about the GRADE.

Thank you for your attention to these details, and I look forward to receiving the revised manuscript.

Best wishes,
Philipp Baumert


[Note: HTML markup is below. Please do not edit.

Reviewers' comments:

Reviewer's Responses to Questions

**Comments to the Author**

1. If the authors have adequately addressed your comments raised in a previous round of review and you feel that this manuscript is now acceptable for publication, you may indicate that here to bypass the “Comments to the Author” section, enter your conflict of interest statement in the “Confidential to Editor” section, and submit your "Accept" recommendation.

Reviewer #2: All comments have been addressed

Reviewer #3: (No Response)

2. Is the manuscript technically sound, and do the data support the conclusions?

The manuscript must describe a technically sound piece of scientific research with data that supports the conclusions. Experiments must have been conducted rigorously, with appropriate controls, replication, and sample sizes. The conclusions must be drawn appropriately based on the data presented.

Reviewer #2: Yes

Reviewer #3: Partly

Author response: We had described the result and concluded in supportive manner

3. Has the statistical analysis been performed appropriately and rigorously?

Reviewer #2: Yes

Reviewer #3: Yes

4. Have the authors made all data underlying the findings in their manuscript fully available?

The [PLOS Data policy](http://www.plosone.org/static/policies.action#sharing) requires authors to make all data underlying the findings described in their manuscript fully available without restriction, with rare exception (please refer to the Data Availability Statement in the manuscript PDF file). The data should be provided as part of the manuscript or its supporting information, or deposited to a public repository. For example, in addition to summary statistics, the data points behind means, medians and variance measures should be available. If there are restrictions on publicly sharing data—e.g. participant privacy or use of data from a third party—those must be specified.

Reviewer #2: Yes

Reviewer #3: No

Author response: all data is described in the manuscript and supported with supplementary data in the revised manuscript

5. Is the manuscript presented in an intelligible fashion and written in standard English?

PLOS ONE does not copyedit accepted manuscripts, so the language in submitted articles must be clear, correct, and unambiguous. Any typographical or grammatical errors should be corrected at revision, so please note any specific errors here.

Reviewer #2: Yes

Reviewer #3: Yes

6. Review Comments to the Author

Please use the space provided to explain your answers to the questions above. You may also include additional comments for the author, including concerns about dual publication, research ethics, or publication ethics. (Please upload your review as an attachment if it exceeds 20,000 characters)

Reviewer #2: Thank you for you point-by-point response.
All comments and suggestions have been adequately addressed by the authors.

Reviewer #3: Dear Editor and Authors!
Thank you for the opportunity to review the paper again.
I believe that the use of GRADE has added robustness to the results obtained.
Here are a few suggestions to include in the paper.

118 Searching strategies

Author response: we had all the comments under searching strategies. See the searching strategies section.

119 The indexed traditional database (Medline/PubMed, Scopus, Web of Science, and Cochrane

Author response: We had corrected as per your comments
120 Library Google Scholar) – Google Scholar isn’t an indexed database.

Author response: thank you we had corrected under grey literature data base

132 strategy design for Embase Cochrane – put coma between Embase and Cochrane

Author response: Thank you we had corrected comma

137 The first search through PubMed, Cochran Library – please, correct Cochran”e”

Author response: Thank you we had corrected
161 Quality appraisal of included studies
162 The articles searched in the database were collected and duplicate articles were removed
163 manually using EndNote (version 7). This sentence can be deleted – is duplicate – line 151 and 152.

Author response: We had removed the text as per your comment.
169 ... After the critical appraisal, the reviewers decided to include or exclude screened articles
170 based on the overall quality of the appraisal score out of 9. The article was prone to exclude
171 when the score was below average, which is of three independent reviewers. –

Authors Response (R1): thank you reviewer this is problem write up it is not due methodological quality rather studies due to the low quality appraisal score were excluded in the analysis of the study.

Review reply: Since no studies were excluded because of low quality (which is methodologically inappropriate), I suggest removing this sentence to avoid confusion.

Review reply 2 – To avoid confusion, I strongly recommend removing this sentence.

Author Response: thank you we had cited the articles removed from the analysis based on quality checks See page 7 Line 178 and the supplementary file for quality checks

Additional suggestions:
Since the authors used GRADE, I suggest including it in the abstract (methods and results). In addition, I suggest rewording the conclusion (of the article and the abstract) to emphasize that the results should be interpreted with caution due to the low certainty of evidence indicated by GRADE.

Author Response: Whe had put the result with text and supplementary file. See page 9 line 223-2232 page: 14 line 336-351 the detail on result section. We also stated on the abstract section about the GRADE.

7. PLOS authors have the option to publish the peer review history of their article ([what does this mean?](https://journals.plos.org/plosone/s/editorial-and-peer-review-process#loc-peer-review-history)). If published, this will include your full peer review and any attached files.


If you choose “no”, your identity will remain anonymous but your review may still be made public.


**Do you want your identity to be public for this peer review?** For information about this choice, including consent withdrawal, please see our [Privacy Policy](https://www.plos.org/privacy-policy).

Reviewer #2: **Yes:** Giuseppe Potrick Stefani

Reviewer #3: No

[NOTE: If reviewer comments were submitted as an attachment file, they will be attached to this email and accessible via the submission site. Please log into your account, locate the manuscript record, and check for the action link "View Attachments". If this link does not appear, there are no attachment files.]

While revising your submission, please upload your figure files to the Preflight Analysis and Conversion Engine (PACE) digital diagnostic tool, <https://pacev2.apexcovantage.com/>. PACE helps ensure that figures meet PLOS requirements. To use PACE, you must first register as a user. Registration is free. Then, login and navigate to the UPLOAD tab, where you will find detailed instructions on how to use the tool. If you encounter any issues or have any questions when using PACE, please email PLOS at <a href="mailto:figures@plos.org">figures@plos.org. Please note that Supporting Information files do not need this step.
